# Response Characteristics of Key Filtering Parameters and Applicability of Interference Detection for Integrated Navigation Under Spoofing Interference

**DOI:** 10.3390/s26175491

**Published:** 2026-08-29

**Authors:** Shiyao Zhao, Jun Fu, Bao Li, Pengfei Jiang

**Affiliations:** College of Electrical Engineering, Naval University of Engineering, Wuhan 430033, China; m24181103@nue.edu.cn (S.Z.);

**Keywords:** GNSS spoofing interference, INS/GNSS integrated navigation, Kalman filter, filtering innovation, IMU accuracy grade

## Abstract

To address Global Navigation Satellite System (GNSS) spoofing threats to Inertial Navigation System (INS)/GNSS integrated navigation systems, this paper analyzes the internal error propagation mechanisms and quantifies perturbation patterns within the Kalman filter (KF) architecture. Mathematical models for step-type, linear ramp, and nonlinear smooth ramp spoofing are established, and the Anomaly Signal-to-Noise Ratio (ASNR) is adopted to quantify disturbances across four core filtering dimensions based on real-world vehicular test data. The results demonstrate that filtering innovations at the forefront of information fusion respond most directly and sensitively (peaking at an ASNR of 341.4181) with a standard zero-mean Gaussian baseline, serving as the optimal metric for spoofing detection; in contrast, error states exhibit marked amplitude attenuation (maximum ASNR of 116.3291), while filter gains and state covariance show negligible variations (maximum ASNRs of 22.6023 and 19.3014, respectively). Further evaluation of Inertial Measurement Unit (IMU) accuracy constraints reveals that under step spoofing, position innovations remain robust (ASNR: 260–510), whereas velocity innovation ASNR drops by approximately 50% with IMU degradation; under linear ramp spoofing, velocity innovations dominate the response (ASNR: 41.16–70.46) while position innovations decay markedly; and under nonlinear smooth ramp spoofing, overall innovations are suppressed, and low-grade IMUs suffer severe noise masking (peak horizontal ASNRs dropping below 10), significantly enhancing attack stealthiness. The findings provide quantitative empirical evidence and theoretical guidance for anti-spoofing design in integrated navigation.

## 1. Introduction

GNSS civil signals inherently possess low power and publicly available code structures, rendering them vulnerable to intentional malicious interference in complex electromagnetic environments [1,2]. Among such threats, spoofing interference generates counterfeit satellite signals that closely match the parameters of authentic signals. This induces the receiver to output erroneous position and velocity estimates without obvious detection. Characterized by high stealthiness and severe destructiveness, spoofing has become one of the primary threats to satellite navigation application security. In recent years, GNSS spoofing incidents have occurred frequently worldwide. For instance, in 2025, spoofing interference caused a tanker collision in the Gulf of Oman; in 2026, widespread regional GNSS spoofing emerged in the Persian Gulf and the Strait of Hormuz, causing the positioning trajectories of over a thousand commercial vessels to deviate severely from their true routes, severely impacting maritime traffic safety and navigational control order.

Currently, the detection of GNSS spoofing interference predominantly relies on a single GNSS data source. These single-source interference detection methods can be broadly categorized into three types: signal baseband processing-based techniques, signal encryption and authentication-based techniques, and Receiver Autonomous Integrity Monitoring (RAIM)-based techniques. When confronted with stealthy, advanced attacks, the aforementioned methods suffer from two insurmountable intrinsic limitations: First, physical limitation constraints. Detection schemes based on signal baseband processing (such as power monitoring, Signal Quality Monitoring (SQM), and Direction of Arrival (DOA) estimation [3,4,5]) rely heavily on the physical characteristics of signals. However, with advancements in Software-Defined Radio (SDR) and high-precision signal synthesis technologies, generative spoofing interference can forge the physical characteristics of authentic signals with high fidelity, drastically degrading the sensitivity of such detection methods or even rendering them ineffective. Second, self-authentication limitation constraints. Although encryption and authentication techniques [6,7] at the navigation message and spreading code layers can achieve identity anti-spoofing, attackers do not need to crack the cryptographic keys; instead, they can invalidate authentication mechanisms simply by rebroadcasting legitimate signals with delayed propagation via meaconing attacks. Meanwhile, data validation techniques based on RAIM [8,9,10] depend on the spatial geometric redundancy among multi-satellite observables. When subjected to coordinated spoofing across all visible satellites or progressive, subtle pull-off spoofing, the spurious observation data still maintain a high degree of mathematical and geometric consistency, thereby causing the internal redundancy verification methods to suffer a holistic judgmental failure.

In comparison, spoofing detection methods based on multi-source fusion have become increasingly prominent and have emerged as a primary research focus among domestic and international scholars in recent years. According to technical approaches, these methods can be categorized into three types: filtering algorithm-based detection, multi-source data consistency-based detection, and artificial intelligence (AI)-based detection [11]. Among these, spoofing interference detection based on INS/GNSS integrated navigation serves as a typical multi-source fusion detection pathway [12,13,14,15,16,17]. This architecture integrates the autonomous and high short-term accuracy merits of the inertial navigation system with the long-term, non-accumulating error advantages of GNSS, thereby achieving complementary performance between the two sensor modalities and standing as the mainstream solution for high-reliability navigation scenarios. From a technical hierarchy perspective, integrated navigation can be classified into loose, tight, and deep integration architectures according to the information fusion depth, and it can be categorized into various technical approaches based on the core fusion algorithm, such as the Kalman filter, $H_{\infty }$ robust filter, and Particle Filter (PF). Among these, the Kalman filter represents the standard fusion scheme with the most mature industrial implementation and the widest application coverage, offering exemplary engineering representativeness. Concurrently, the majority of existing spoofing detection and mitigation studies in integrated navigation are developed upon the Kalman filtering framework; hence, conducting interference impact analysis targeting this framework offers universal academic and engineering value.

In terms of the research on the impact mechanism of spoofing interference on integrated navigation, although existing work [18] has conducted a beneficial discussion on the internal response of the Kalman filter, a deeper analysis reveals two main limitations: First, they lack an in-depth dissection of the internal propagation mechanism of spoofing errors within the KF, failing to conduct fine-grained quantitative analyses across multi-dimensional core parameters such as filter innovation, error states, filter gain, and state covariance. Second, most works are restricted to a single attack mode or a fixed hardware platform, lacking systematic quantitative comparative analyses across diverse operating conditions, such as multiple spoofing pull-off strategies or varying IMU precision grades. To address these issues, this paper takes a representative 15-state, 6-measurement INS/GNSS integrated navigation system as the research object to conduct an in-depth investigation into the impact mechanisms and quantitative characteristics of spoofing interference.

The core research contributions of this paper are summarized as follows: First, three types of spoofing interference models—step-type, linear ramp-type, and nonlinear ramp-type—are constructed. The impulse response characteristics of different pull-off strategies are systematically compared across four dimensions: filter innovation, error states, filter gain, and state covariance, with the Anomaly Signal-to-Noise Ratio (ASNR) metric introduced to quantitatively characterize the system perturbation level. On this basis, the constraint laws of IMU precision on the system innovation response characteristics are further revealed, providing theoretical foundation and empirical data support for subsequent research on spoofing interference detection in integrated navigation systems.

## 2. Modeling of Typical GNSS Spoofing Interference

GNSS spoofing interference misleads the receiver into locking onto counterfeit signals by transmitting pseudo-satellite signals that closely match the structure and parameters of authentic satellite signals, thereby outputting erroneous position and velocity solutions. The resulting systematic biases enter the integrated navigation system as measurement errors, ultimately corrupting the overall navigation state estimation. Depending on the spoofing strategy employed, typical spoofing interference can be categorized into two classes: step-type spoofing and ramp-type spoofing [19], which exhibit distinct differences in error evolution dynamics and stealthiness.

### 2.1. Step-Type Position Spoofing Model

Step-type spoofing is the most fundamental spoofing interference mode, characterized by an instantaneous injection of a fixed position bias $P_{step}$ into the GNSS observations at the onset time $t_{s}$. The position bias model for step-type spoofing can be expressed as:

(1) $$\Delta P_{step}(t)=\left\{\begin{array}{ll} 0, & t<t_{s}\\ P_{step}, & t\geq t_{s} \end{array}\right.$$

In practical engineering, after the spoofing signal induces a sudden jump in the receiver position, the trajectory and velocity estimation output by the receiver depend on the generation strategy of the spoofer:

If the spoofing signal merely locks the position to a fixed virtual point, the receiver’s output position remains constant after time $t_{s}$, and the output velocity degrades to zero. In this case, the measurements injected into the filter are:

(2) $$\left\{\begin{array}{l} {\tilde{P}}_{GNSS}(t)=P_{true}(t_{s})+P_{step}\\ {\tilde{V}}_{GNSS}(t)=0 \end{array}\right.$$

If the spoofer fails to perceive the vehicle’s maneuvering and merely broadcasts a constant velocity parameter $V_{const}$ to the receiver, the output position extrapolates linearly along this velocity direction starting from the jump point. In this case, the measurements injected into the filter are:

(3) $$\;\left\{\begin{array}{l} {\tilde{P}}_{GNSS}(t)=P_{true}(t_{s})+P_{step}+V_{const}(t-t_{s})\\ {\tilde{V}}_{GNSS}(t)=V_{const} \end{array}\right.$$

If the spoofer can track the vehicle’s motion state in real time and maintain relative rest between the spoofing signal and the vehicle, the output position undergoes an overall translation on the basis of the true trajectory, while the estimated velocity remains consistent with the vehicle’s true velocity. In this case, the measurements injected into the filter are:

(4) $$\left\{\begin{array}{l} {\tilde{P}}_{GNSS}(t)=P_{true}(t_{s})+P_{step}\\ {\tilde{V}}_{GNSS}(t)=V_{true}(t) \end{array}\right.$$

where $P_{true}(t)$ and $V_{true}(t)$ denote the vehicle’s true real-time position and velocity, respectively; $P_{true}(t_{s})$ represents the vehicle’s true position at the onset time of spoofing.

The bias of this type of spoofing exhibits instantaneous step characteristics. Upon activation of the interference, the position output by the GNSS immediately generates a systematic bias of a fixed magnitude. Directly injected into the integrated navigation filtering framework as an observation error, this bias exhibits marked differences from normal observation noise and routine vehicle maneuvering errors, resulting in relatively weak overall stealthiness.

### 2.2. Linear Ramp-Type GNSS Spoofing Model

Linear ramp pull-off is a fundamental ramp spoofing strategy. Its essence lies in the spoofer decoupling from the vehicle’s true maneuvering and directly presetting a constant velocity vector $V_{ramp}$ for the receiver, causing the position solved by the receiver to linearly extrapolate and deviate over time starting from the spoofing onset.

Let $t_{s}$ denote the spoofing onset time. During the ongoing spoofing stage ($t\geq t_{s}$), the spurious GNSS position and velocity measurement equations can be expressed as:

(5) $$\left\{\begin{array}{l} {\tilde{P}}_{GNSS}(t)=P_{true}(t_{s})+V_{ramp}\cdot (t-t_{s})\\ {\tilde{V}}_{GNSS}(t)=V_{ramp} \end{array}\right.$$

This model achieves a continuous and smooth pull-off in position measurements starting from the onset. However, at the spoofing onset time $t_{s}$, the velocity measurement abruptly jumps from the true value $V_{true}(t_{s})$ at the current instant to the preset value $V_{ramp}$, producing a step change in the velocity domain. Consequently, its stealthiness in the velocity domain remains relatively weak.

### 2.3. Nonlinear Ramp-Type GNSS Spoofing Model

With the evolution of spoofing attack techniques, advanced spoofers often employ nonlinear smooth pull-off strategies to achieve high-stealthiness spoofing. Although conventional linear ramp pull-off achieves a gradual diversion in position, abrupt velocity jumps occur at the onset and termination instants of the attack, substantially diminishing the stealthiness of the spoofing signal.

To simulate extreme spoofing scenarios that are more threatening and closely representative of advanced practical attack techniques, a nonlinear smooth spoofing model based on a half-cosine transition is constructed.

Let $P_{smooth}$ denote the target pull-off position bias vector preset by the spoofer, representing the maximum offset displacement applied relative to the vehicle’s initial spoofed position upon the completion of the attack. The real-time position and velocity observation deviations induced by the spoofing are denoted as $\Delta P_{smooth}(t)$ and $\Delta V_{smooth}(t)$, respectively. To achieve a smooth and continuous pull-off process without sudden jumps, a dimensionless piecewise half-cosine smooth amplitude modulation coefficient β(t) is introduced, allowing the real-time position and velocity observation deviations to be expressed as:

(6) $$\left\{\begin{array}{l} \Delta P_{smooth}(t)=\beta (t)\cdot P_{smooth}\\ \Delta V_{smooth}(t)=\dot{\beta }(t)\cdot P_{smooth} \end{array}\right.$$

The spurious GNSS position and velocity measurement equations can then be formulated as:

(7) $$\left\{\begin{array}{l} {\tilde{P}}_{GNSS}(t)=P_{true}(t_{s})+\Delta P_{smooth}(t)\\ {\tilde{V}}_{GNSS}(t)=V_{true}(t_{s})+\Delta V_{smooth}(t) \end{array}\right.$$

where $\dot{\beta }(t)$ is the velocity modulation coefficient, and $t_{s}$ denotes the spoofing onset time. Setting the ramp termination time as $t_{e}$ (with the transition duration defined as $T=t_{e}-t_{s}$), the mathematical definition of the piecewise smooth coefficient β(t) is given by:

(8) $$\beta (t)=\left\{\begin{array}{ll} 0, & t<t_{s}\\ \frac{1}{2}\left[1-\cos\left(\pi \cdot \frac{t-t_{s}}{T}\right)\right], & t_{s}\leq t<t_{e}\\ 1, & t\geq t_{e} \end{array}\right.$$

Taking the first-order derivative of β(t) with respect to time t yields the matched velocity modulation coefficient $\dot{\beta }(t)$:

(9) $$\dot{\beta }(t)=\left\{\begin{array}{ll} 0, & t<t_{s}\\ \frac{\pi }{2T}\sin\left(\pi \cdot \frac{t-t_{s}}{T}\right), & t_{s}\leq t<t_{e}\\ 0, & t\geq t_{e} \end{array}\right.\;$$

**Figure 1:**
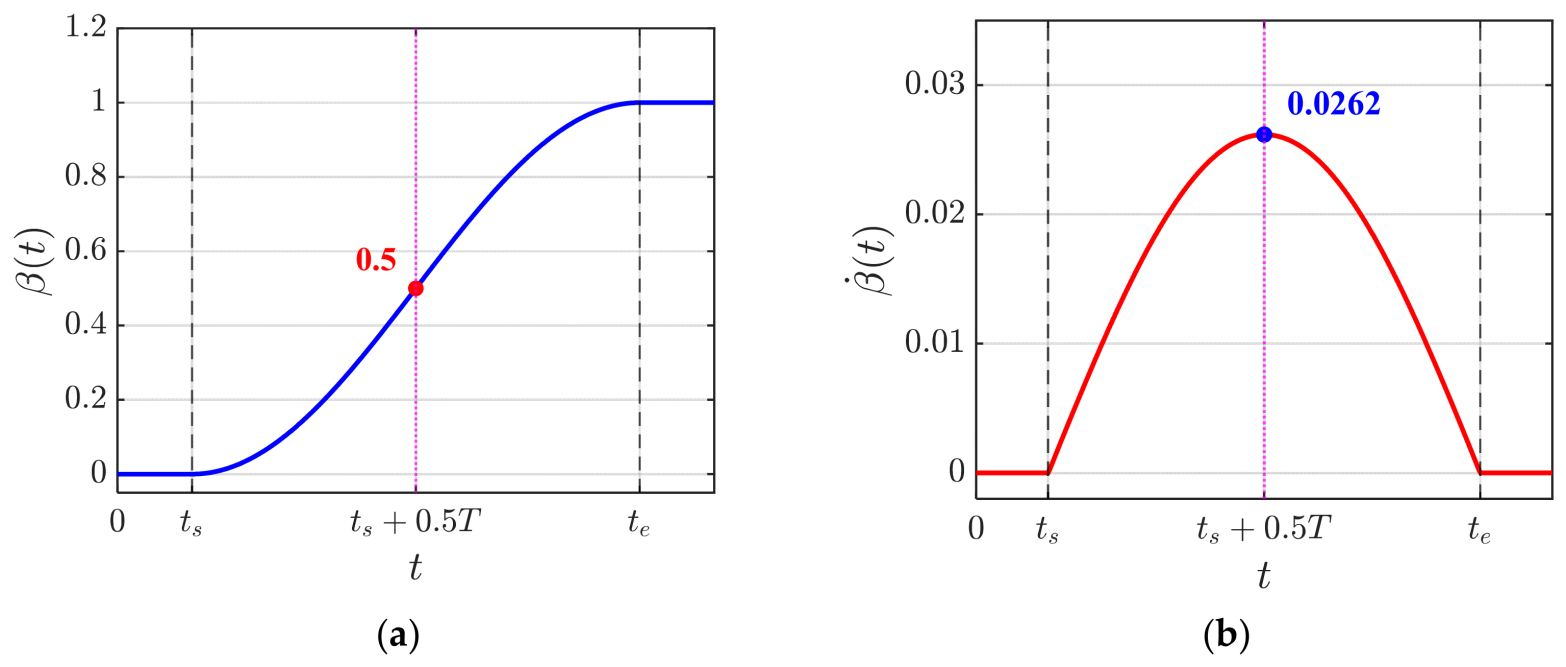
Curves of modulation coefficients: (**a**) Position smooth modulation coefficient β(t); (**b**) Velocity modulation coefficient $\dot{\beta }(t)$.

Taking the pull-off duration $T_{u}=60\;s$ as an example, the variation curves of the modulation coefficient β(t) and its first derivative $\dot{\beta }(t)$ are shown in Figure 1.

As can be observed, the position and velocity in the aforementioned half-cosine ramp spoofing model maintain global continuity, thereby avoiding step discontinuities in either position or velocity at the onset of spoofing.

## 3. Propagation Mechanism and Evolution Dynamics of GNSS Spoofing Interference Within Integrated Navigation Systems

Integrated navigation systems achieve high-precision corrections of navigation parameters—such as vehicle attitude, velocity, and position—by combining the complementary strengths of INS and GNSS. The fundamental analytical framework in this study adopts a canonical 15-state, 6-measurement INS/GNSS integration model. The system utilizes a KF to estimate the core error quantities of the INS as the state vector. The 15-dimensional error state vector of the system is expressed as [20]:

(10) $$X={\left[\phi \;\;\;\delta V_{\mathrm{INS}}\;\;\;\delta P_{\mathrm{INS}}\;\;\;\varepsilon \;\;\;b\right]}^{T}$$

where $\phi ={\left[\phi_{E}\;\phi_{N}\;\phi_{U}\right]}^{T}$, $\delta V_{\mathrm{INS}}={\left[\delta V_{E}\;\delta V_{N}\;\delta V_{U}\right]}^{T}$, and $\delta P_{\mathrm{INS}}={\left[\delta \lambda \;\delta L\;\delta h\right]}^{T}$ denote the attitude error, velocity error, and position error vectors of the INS, respectively; ε represents the gyro drift vector; and b denotes the accelerometer bias vector.

The system measurement vector $Z_{k}$ is constructed from the velocity and position differences between the INS and GNSS outputs, which is formulated as:

(11) $$Z_{k}=\left[\begin{array}{c} V_{\mathrm{INS},E,k}-V_{\mathrm{GNSS},E,k}\\ V_{\mathrm{INS},N,k}-V_{\mathrm{GNSS},N,k}\\ V_{\mathrm{INS},U,k}-V_{\mathrm{GNSS},U,k}\\ \lambda_{\mathrm{INS},k}-\lambda_{\mathrm{GNSS},k}\\ L_{\mathrm{INS},k}-L_{\mathrm{GNSS},k}\\ h_{\mathrm{INS},k}-h_{\mathrm{GNSS},k} \end{array}\right]$$

where $v_{INS,E,k},v_{INS,N,k},v_{INS,U,k},\lambda_{INS,k},L_{INS,k},h_{INS,k}$ are the east-north-up velocities and geodetic coordinates (longitude, latitude, altitude) derived from the INS mechanization; $v_{GNSS,E,k},v_{GNSS,N,k},v_{GNSS,U,k},\lambda_{GNSS,k},L_{GNSS,k},h_{GNSS,k}$ denote the corresponding velocity and position information provided by the GNSS receiver.

To accommodate the requirements of digital filtering, the continuous-time system is discretized. The unified discrete state propagation equation and measurement equation are given by:

(12) $$\left\{\begin{array}{l} X_{k}=\Phi_{k|k-1}X_{k-1}+\Gamma_{k-1}W_{k-1}\\ Z_{k}=H_{k}X_{k}+V_{k} \end{array}\right.\;$$

where k denotes the discrete filtering epoch; $\Phi_{k|k-1}$ is the discrete state transition matrix from epoch k−1 to k; $\Gamma_{k-1}$ is the process noise driving matrix; $H_{k}$ is the measurement matrix; $W_{k-1}$ and $V_{k}$ are mutually uncorrelated zero-mean Gaussian white noise sequences with covariance matrices denoted by the process noise covariance $Q_{k-1}$ and measurement noise covariance $R_{k}$, respectively.

Based on the established INS/GNSS discrete-time state and measurement models, the Kalman filter achieves optimal state estimation through the alternating recursion of time update and measurement update. The time update phase predicts the current system state using the prior state estimate and covariance from the preceding epoch, formulated as:

(13) $$\left\{\begin{array}{l} {\hat{X}}_{k|k-1}=\Phi_{k|k-1}{\hat{X}}_{k-1}\\ P_{k|k-1}=\Phi_{k|k-1}P_{k-1}\Phi_{k|k-1}^{T}+\Gamma_{k-1}Q_{k-1}\Gamma_{k-1}^{T} \end{array}\right.\;$$

where ${\hat{X}}_{k-1}$ and $P_{k-1}$ denote the posterior state estimate vector and posterior error covariance matrix at epoch k−1, respectively; ${\hat{X}}_{k|k-1}$ and $P_{k|k-1}$ are the one-step predicted state estimate and prior error covariance matrix from epoch k−1 to k.

Upon acquiring the measurement information at the current epoch, the filter executes the measurement update to correct the predicted values:

(14) $$\left\{\begin{array}{l} \delta Z_{k}=Z_{k}-H_{k}{\hat{X}}_{k|k-1}\\ K_{k}=P_{k|k-1}H_{k}^{T}{\left(H_{k}P_{k|k-1}H_{k}^{T}+R_{k}\right)}^{-1}\\ {\hat{X}}_{k}={\hat{X}}_{k|k-1}+K_{k}(Z_{k}-H_{k}{\hat{X}}_{k|k-1})\\ P_{k}=(I-K_{k}H_{k})P_{k|k-1} \end{array}\right.\;$$

where $\delta Z_{k}$ is the filter innovation; $K_{k}$ is the Kalman gain matrix; ${\hat{X}}_{k}$ and $P_{k}$ are the corrected posterior state estimate vector and posterior error covariance matrix at the current epoch; and I is the identity matrix matching the dimension of the state vector.

**Figure 2:**
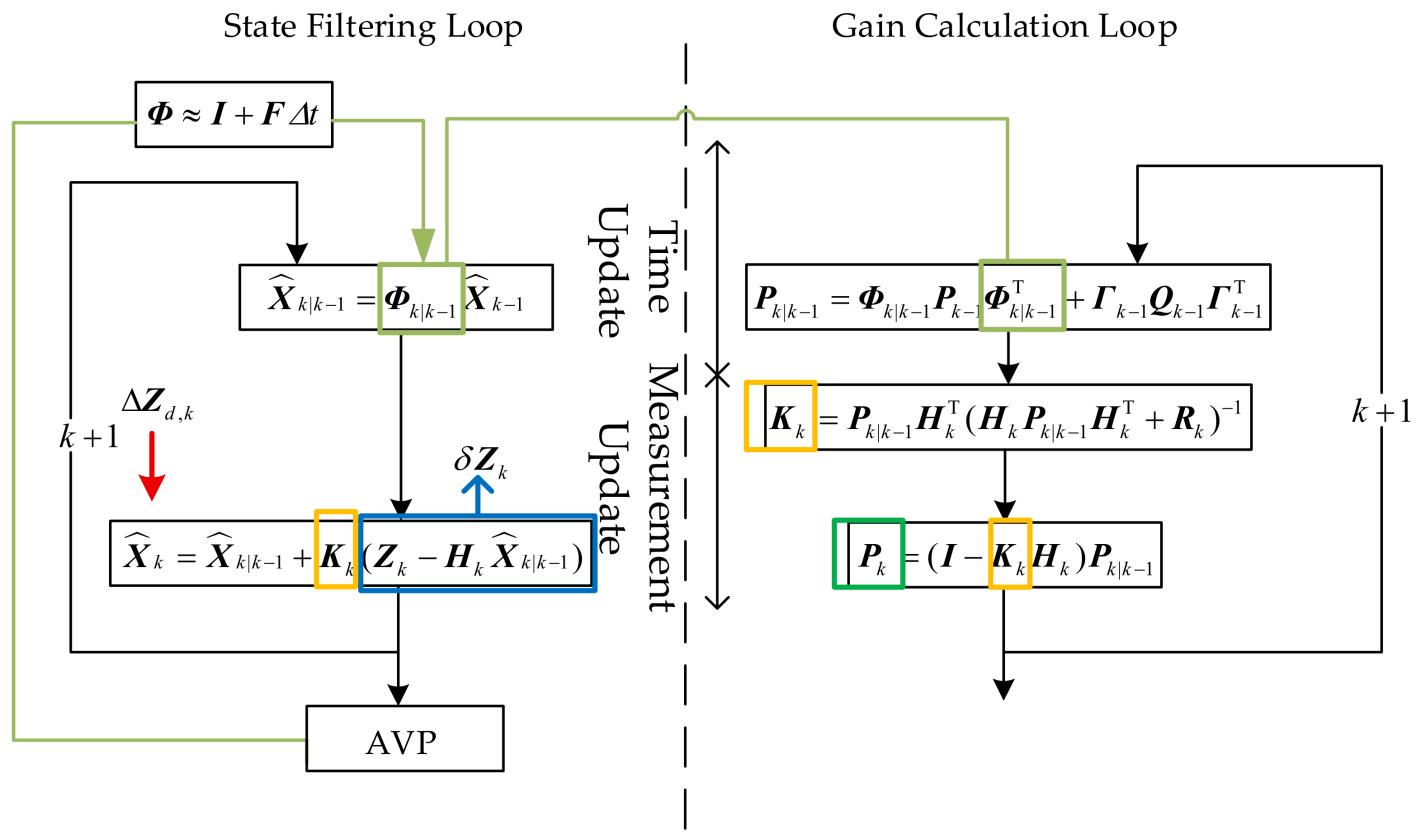
Standard Kalman filter with introduced spoofing interference.

When the integrated navigation system is subjected to GNSS spoofing attacks, spurious radio frequency signals transmitted by the spoofer induce the GNSS receiver to resolve erroneous position and velocity outputs containing malicious biases. Consequently, the actual measurement vector $Z_{k}$ contaminated by spoofing interference can be formulated as:

(15) $$Z_{k}=H_{k}X_{k}+V_{k}-\Delta Z_{d,k}$$

where $\Delta Z_{d,k}$ is the spoofing interference injection vector at epoch $t_{k}$. The recursive loop architecture of the standard discrete Kalman filter and the injection mechanism of spoofing interference are illustrated in Figure 2.

As indicated by the internal logical loops and recursive equations in Figure 2, the impact of GNSS spoofing interference on the integrated navigation system exhibits an inside-out cascading propagation characteristic. Once the spoofing bias $\Delta Z_{d,k}$ penetrates the system, it directly corrupts the filter innovation $\delta Z_{k}$ during the measurement update phase. The contaminated innovation is subsequently weighted and mapped via the Kalman gain $K_{k}$, directly inducing systematic biases in the posterior error state estimate ${\hat{X}}_{k}$. In a closed-loop integration architecture, these corrupted error state estimates are fed back in real time to the INS mechanization loop to enforce erroneous corrections on core navigation parameters—including attitude, velocity, position, and sensor biases. Since the system’s discrete state transition matrix Φ is highly dependent on real-time navigation states and attitude projection baselines, this distorted closed-loop feedback correction indirectly triggers deformations in the state transition matrices of subsequent epochs, gradually perturbing the temporal evolution of the Kalman gain $K_{k}$ and the error covariance matrix $P_{k}$. The specific quantitative perturbation patterns across different state dimensions under various spoofing modes and parameters will be thoroughly dissected in the subsequent simulation experiments.

## 4. Simulation Analysis of GNSS Spoofing Interference Impacts on Integrated Navigation

### 4.1. Construction of Typical GNSS Spoofing Scenarios

To comprehensively evaluate the error response characteristics of the integrated navigation system under different spoofing strategies, this chapter employs the built-in data_cpt vehicular field dataset from the open-source GINav integrated navigation platform as the data source [21]. The entire duration of this dataset is 1720 s. Three typical GNSS spoofing scenarios—step-type, linear ramp-type, and nonlinear ramp-type—are simulated within the time interval of 600 s to 660 s.

For the step-type scenario, in order to investigate the coupling effect of position steps on velocity during the evaluation, it is assumed that the spoofing signal solely injects step tampering into the GNSS position information, while the velocity observations remain consistent with the vehicle’s actual velocity. For the ramp-type scenarios, two conditions—linear and nonlinear ramps—are configured. The linear ramp spoofing directly presets a constant velocity in the GNSS velocity observations. In contrast, the nonlinear ramp spoofing adopts a half-cosine smooth transition model to implement continuous position pull-off, with the velocity observations superimposed with nonlinear time-varying velocity biases determined by the time derivative of position, thereby achieving high-order continuous smooth transitions in both position and velocity within the time domain.

The simulation parameters for the spoofing interference scenarios are listed in Table 1.

It should be noted that, through kinematic extrapolation, the terminal target point of linear ramp-type spoofing under these parameters reaches a substantial horizontal displacement of 637.10 m (430.04 m in the East and 470.06 m in the North) over the 60 s duration, resulting from the superposition of the vehicle’s initial velocity and the injected bias. Considering that such a jump magnitude is excessively large and that step-type and ramp-type spoofing possess fundamentally distinct temporal dynamics (instantaneous abrupt jump versus continuous gradual pull-off) that preclude strict one-to-one controlled variable comparison across the entire time domain, a moderate step magnitude of 100 m is configured as the baseline benchmark in this study. On this basis, to scientifically evaluate the stealthiness mechanisms between different pull-off strategies, the two ramp-type spoofing models strictly adhere to the controlled variable principle of “equivalent total displacement,” ensuring that both scenarios achieve an identical cumulative displacement ΔP within the same duration, satisfying:

(16) $$P_{smooth}=P_{ramp}=\Delta P$$

Through derivation, a rigorous mapping relationship is established between the peak bias velocity $V_{\max}$ of nonlinear ramp spoofing and the total displacement ΔP:

(17) $$V_{\max}=\dot{\beta }(T_{u}/2)\cdot \Delta P$$

**Figure 3:**
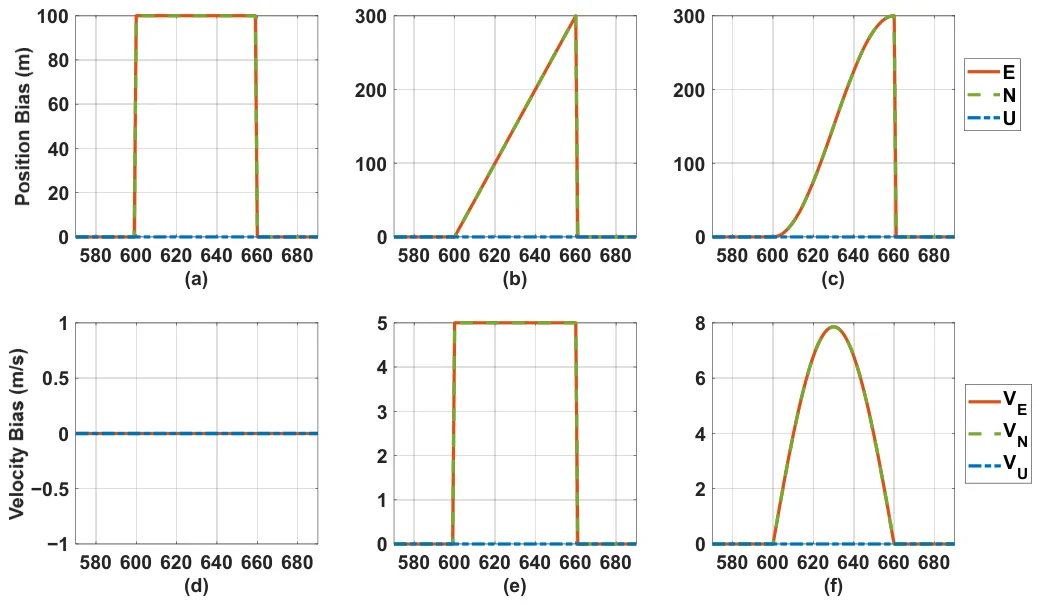
Time-series curves of injected position and velocity biases: (**a**) Position bias of step spoofing; (**b**) Position bias of linear ramp spoofing; (**c**) Position bias of nonlinear ramp spoofing; (**d**) Velocity bias of step spoofing; (**e**) Velocity bias of linear ramp spoofing; (**f**) Velocity bias of nonlinear ramp spoofing.

Taking the instant $t_{s}$ as the reference baseline, the position and velocity biases under different spoofing modes are shown in Figure 3. As observed in Figure 3, Figure 3a,d illustrate the characteristics of step spoofing, where instantaneous position jumps occur, inducing extremely large pulse-like position biases at the injection instant; Figure 3b,e demonstrate the characteristics of linear ramp spoofing, where the position bias exhibits linear first-order growth corresponding to an injected velocity with a constant step profile; Figure 3c,f depict the characteristics of nonlinear ramp spoofing, where the position bias undergoes a smooth nonlinear transition and the corresponding velocity bias presents a continuous bell-shaped curve, exhibiting exceptionally high stealthiness.

Regarding system noise modeling, to avoid interference from high vehicle dynamics in evaluating the intrinsic noise of inertial sensors, this paper does not perform unselective statistical estimation over the entire 1720 s dataset. Instead, relying on a high-precision offline reference solution, a strict velocity threshold (V<0.3 m/s) is set to accurately filter observation segments where the vehicle is stationary or in a stable low-speed state. For the selected stationary sequence, Allan variance theory is employed to quantitatively calibrate the broadband white noise of the IMU in the time domain.

Let the raw stationary IMU sampling sequence be divided into k non-overlapping clusters based on a smoothing time window τ. If the observation average within the k-th cluster is denoted by ${\bar{y}}_{k}(\tau )$, the Allan variance $\sigma^{2}(\tau )$ at this time scale τ is defined as:

(18) $$\sigma^{2}(\tau )=\frac{1}{2(K-1)}\sum_{k=1}^{K-1}{\left[{\bar{y}}_{k+1}(\tau )-{\bar{y}}_{k}(\tau )\right]}^{2}$$

According to inertial sensor error mechanisms, on the log-log characteristic curve of Allan standard deviation, the high-frequency random walk (gyroscope Angle Random Walk, ARW/accelerometer Velocity Random Walk, VRW) induced by broadband white noise integration exhibits a characteristic slope of −1/2, and its theoretical expression is:

(19) $$\sigma (\tau )=\frac{N}{\sqrt{\tau }}$$

where N is the random walk coefficient. To extract this parameter, a smoothing time of τ=1 s is directly selected during data preprocessing, as the Allan standard deviation intercept σ(1) at this scale is mathematically strictly equivalent to the random walk coefficient N. For IMU data with a sampling frequency of 100 Hz, a sliding average window with a length of 1 s (i.e., 100 epochs) is directly constructed, and the sliding average is subtracted from the raw observation sequence, thereby effectively eliminating low-frequency bias instability and slow thermal drift. Taking the robust standard deviation of the retained high-frequency residual sequence is equivalent to extracting the Allan standard deviation σ(1) at τ=1 s, which accurately calibrates the random walk parameters required for the process noise matrix (Q) of the integrated navigation Kalman filter. Furthermore, by statistically calculating the differences between raw GNSS measurements and the reference solution under interference-free conditions, the noise standard deviations for positioning and velocity measurements are obtained, providing an objective physical basis for the measurement noise matrix (R). The finalized core sensor configuration parameters are listed in Table 2.

**Figure 4:**
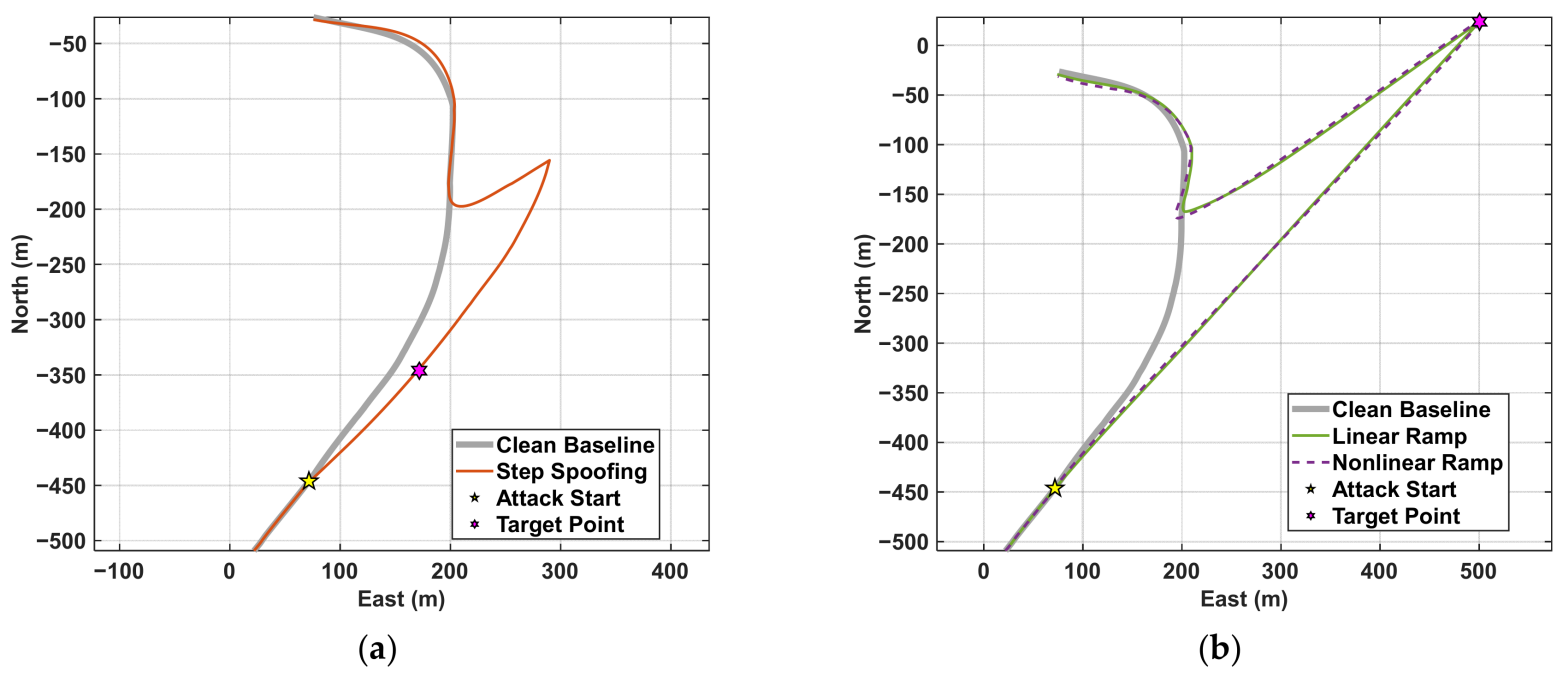
Global trajectory comparison maps of spoofing simulation scenarios: (**a**) 2D horizontal trajectory of step-type spoofing; (**b**) 2D horizontal trajectory of ramp-type spoofing.

To further clarify the spatial relative relationship of the aforementioned spoofing parameters in practical scenarios, the preset spoofing trajectories and the true reference baseline are mapped globally in Figure 4.

### 4.2. Analysis of the Response Characteristics of Filter Parameters Under Typical Spoofing Scenarios

In the simulation phase, this paper delves into the internal loops of the Kalman filter, focusing on the evolutionary processes across four core dimensions: measurement innovation, filter gain, error states, and state covariance matrix. This deeply reveals the corruption mechanisms imposed by different spoofing strategies on the information fusion process and system integrity of the GNSS/INS integrated navigation system.

#### 4.2.1. Analysis of the Impact of GNSS Spoofing Interference on Filtering Innovation

Filter innovation reflects the difference between the actual system measurements and the one-step predicted state values of the filter, serving as a core indicator to evaluate the consistency between observation data and the system dynamic model. Under ideal, interference-free operating conditions, the filter innovation theoretically follows a zero-mean Gaussian white noise distribution:

(20) $$\delta Z_{k}\sim N\left(0,S_{k}\right)$$

where $S_{k}$ is the theoretical innovation covariance matrix, calculated as:

(21) $$S_{k}=H_{k}P_{k|k-1}H_{k}^{T}+R_{k}$$

where $P_{k|k-1}$ denotes the one-step predicted covariance matrix, and $R_{k}$ is the measurement noise covariance matrix. In the time domain, the innovations across different sampling epochs are mutually independent and uncorrelated, satisfying $E[\delta Z_{k}\delta Z_{j}^{T}]=0\;(k\neq j)$. When the system is subjected to GNSS spoofing attacks, artificially constructed false biases are superimposed onto the measurement vector $Z_{k}$, inducing abrupt perturbations in the innovation and degrading its statistical characteristics from a zero-mean Gaussian distribution to a non-zero-mean distribution.

**Figure 5:**
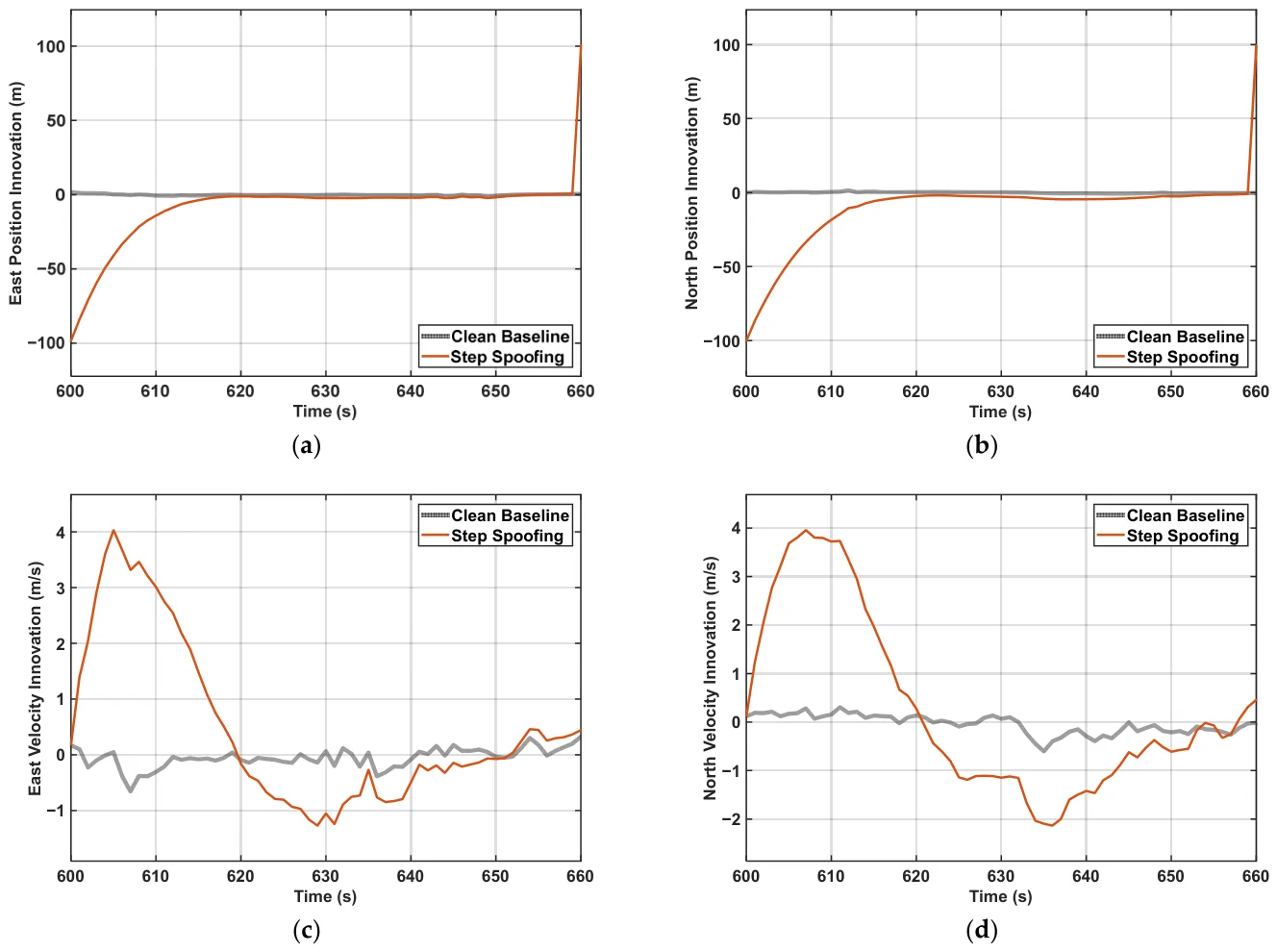
Comparison of horizontal position and velocity filter innovations under the step-type spoofing scenario: (**a**) East position innovation; (**b**) North position innovation; (**c**) East velocity innovation; (**d**) North velocity innovation.

The spoofing strategy designed in this section applies interference along the horizontal direction; thus, the horizontal channels can reflect the impact of spoofing signals on the filtering process most directly and sensitively. To intuitively compare the evolutionary discrepancies of innovations under different spoofing strategies, the position and velocity channel innovations in the east and north directions during the spoofing period are extracted for comparative analysis. First, the step-type spoofing scenario is examined, with the results shown in Figure 5.

As can be observed from Figure 5, at the instant of spoofing injection, the horizontal position innovations exhibit a pulse jump that matches the spoofing configuration with high consistency. Moreover, although the attack is directly applied solely to the position measurements, it synchronously induces severe, large-amplitude oscillations in the velocity innovations due to the system state coupling effects.

**Figure 6:**
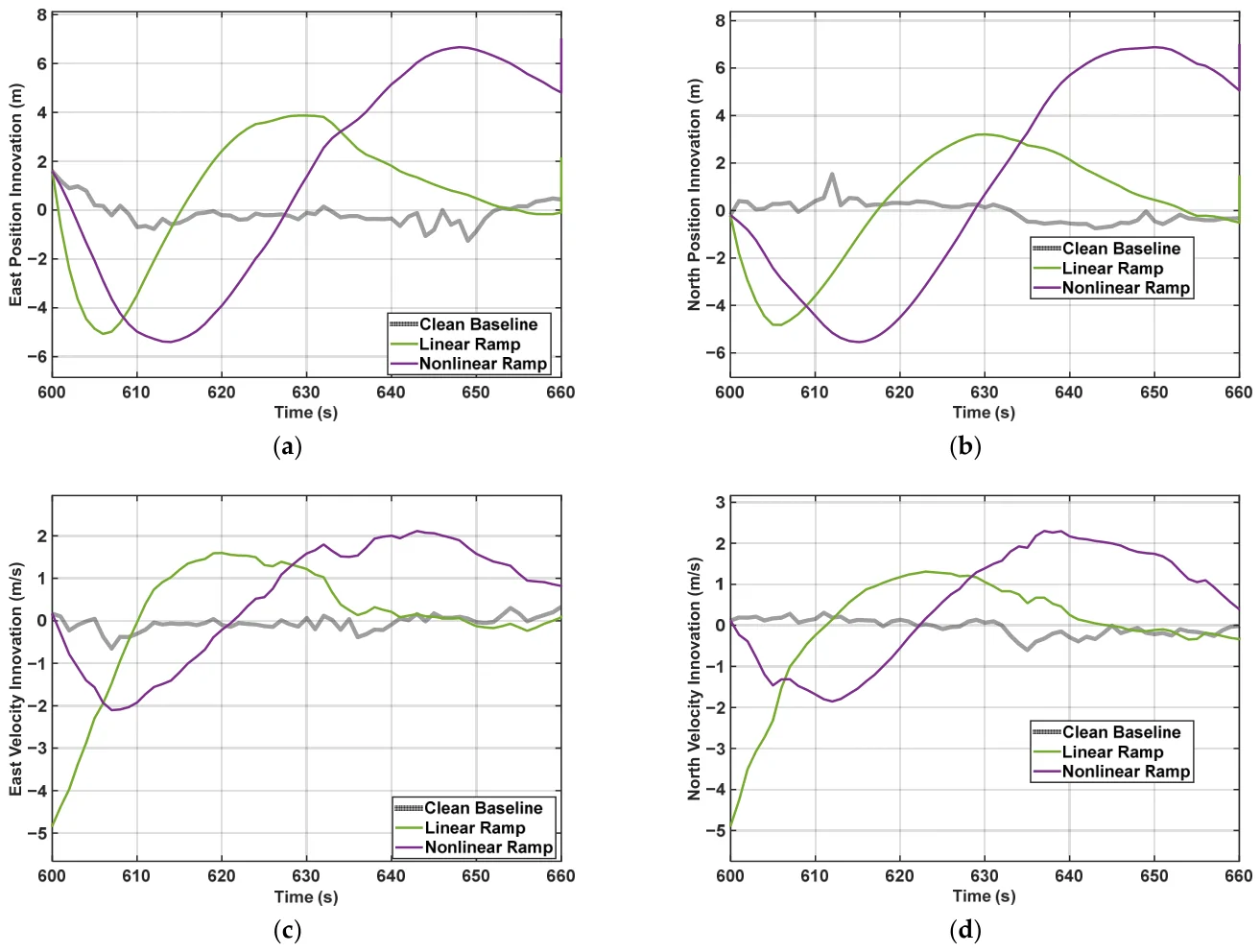
Comparison of horizontal position and velocity filter innovations under ramp-type spoofing scenarios: (**a**) East position innovation; (**b**) North position innovation; (**c**) East velocity innovation; (**d**) North velocity innovation.

Compared to the instantaneous jumps induced by step attacks, ramp-type spoofing strategies exhibit markedly distinct dynamic evolution characteristics in system innovation perturbations. Figure 6 presents the horizontal innovation response curves under the linear and nonlinear ramp spoofing scenarios.

From the response curves, the impact mechanisms and discrepancies of different ramp spoofing strategies on the filter innovation characteristics can be distinctly compared:

1. Linear ramp spoofing: Owing to the constant-velocity ramp pull-off strategy, the position remains continuous at the onset and termination instants of the interference; hence, the position innovations exhibit progressive fluctuations rather than pulse jumps. In the velocity channels, however, because the spurious measurements contain a constant velocity bias, the velocity innovations undergo a direct step-like jump at the onset of interference, though they rapidly converge within a short duration.
2. Nonlinear ramp spoofing: Due to the half-cosine smooth transition, the position and velocity curves remain continuous in the time domain; hence, neither position nor velocity innovations undergo instantaneous jumps at the interference onset. Nevertheless, the entire ramp process still excites significant innovation fluctuations. Furthermore, distinct from the rapid convergence observed in the other two spoofing types, the convergence of innovations under nonlinear ramp spoofing is noticeably slower due to the introduction of time-varying velocity biases.

**Figure 7:**
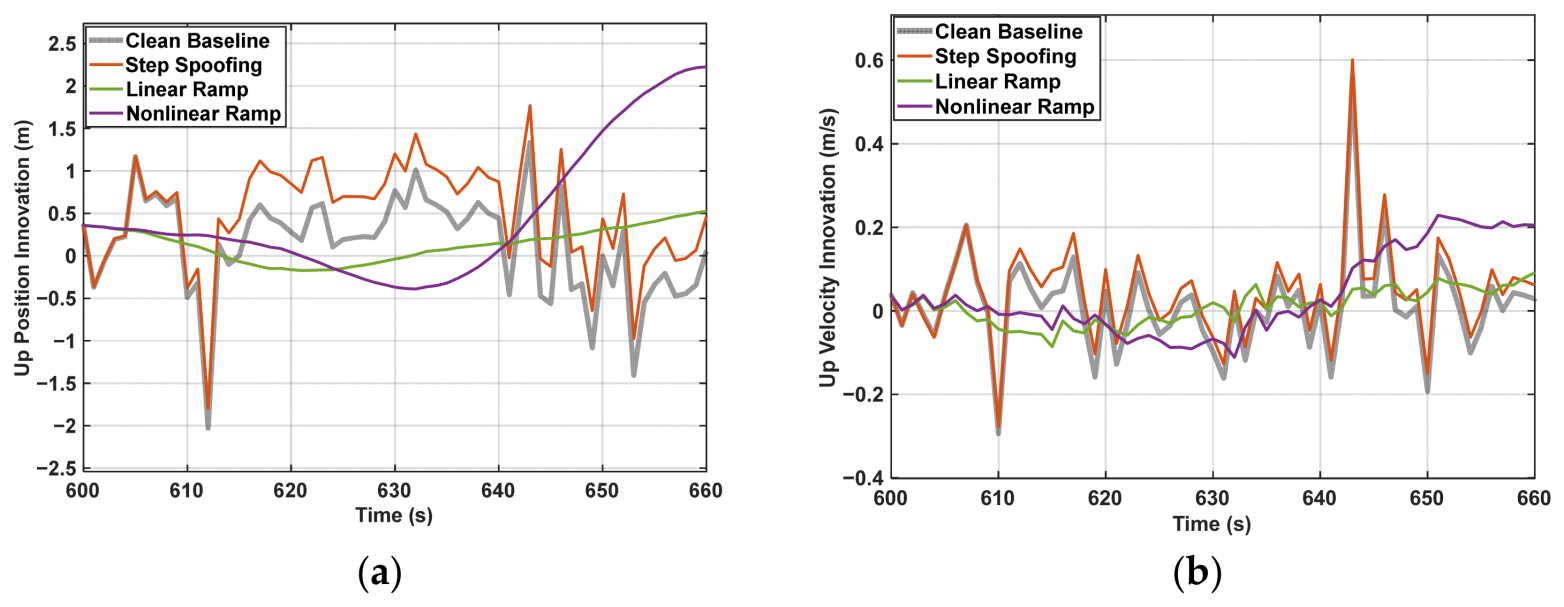
Comparison of up position and velocity filter innovations under different spoofing scenarios: (**a**) Up position innovation; (**b**) Up velocity innovation.

In summary, spoofing attacks—whether transient jumps or smooth transitions—significantly impact both position and velocity innovations. To comprehensively evaluate the spatial perturbation characteristics of spoofing interference, this section further analyzes the innovation variations in the up channels, with the corresponding response curves shown in Figure 7.

As observed from the innovation curves in the up channel, although spoofing is not directly applied along the vertical direction, the up innovation still exhibits slight fluctuations due to the multidimensional cross-state coupling of the Kalman filter. Nonetheless, compared to the horizontal channels, the perturbation level in the vertical channel is considerably smaller, with its overall magnitude remaining within normal bounds.

To further quantitatively evaluate the impact of different spoofing strategies on innovations, the Anomaly Signal-to-Noise Ratio (ASNR) is introduced to quantify the fluctuation degree of innovations. ASNR can intuitively reflect the ratio of the maximum abrupt magnitude by which the perturbed parameter deviates from the normal baseline within the spoofing time window to the baseline noise fluctuation, thereby effectively overcoming the limitation of inconsistent physical dimensions among different parameters. Its calculation formula is:

(22) $$\mathrm{ASNR}=\frac{\max|x^{spoof}(t)-\mu_{clean}|}{\sigma_{clean}},\;\;\;\;t\in [t_{s},t_{e}]$$

where $x{\left(t\right)}^{spoof}$ denotes the actual time-domain response value of the parameter under the spoofing scenario at time t; $\mu_{clean}$ and $\sigma_{clean}$ are the statistical mean and standard deviation of the parameter within the identical time window under the interference-free scenario, respectively. The ASNR indicators of innovation channels under various spoofing scenarios are extracted, and the quantitative comparison results are shown in Table 3.

Synthesizing Table 3 and the aforementioned curve analysis, GNSS spoofing interference imposes significant and direct impacts on filter innovations, exhibiting three typical characteristics:

1. State Cross-Coupling: Position and velocity innovations demonstrate a high degree of correlation in their dynamic responses.
2. Strategy-Dependent Innovation Convergence: When subjected to constant-bias interference, the filter rapidly assimilates the contaminated measurements to force innovation convergence; however, in the presence of the time-varying biases of nonlinear ramp spoofing, innovations are continuously excited and exhibit persistent fluctuations that fail to converge rapidly.
3. Spatial Directionality of Error Propagation: The fluctuation magnitude of innovations is highly correlated with the dimensions into which spoofing is injected, predominantly concentrating in specific interfered directions, whereas the impact on non-interfered directions remains relatively limited.

#### 4.2.2. Analysis of the Impact of GNSS Spoofing Interference on Kalman Filter Gain

When subjected to GNSS spoofing interference, the innovation anomaly $\delta Z_{k}$ directly affects the error state estimates via the Kalman gain $K_{k}$. Based on the physical interpretation of the Kalman gain and the measurement update logic, the gain components in the 15-dimensional system are categorized into three classes for detailed discussion: primary gains, position coupling gains, and velocity coupling gains.

1. Primary Gains

**Figure 8:**
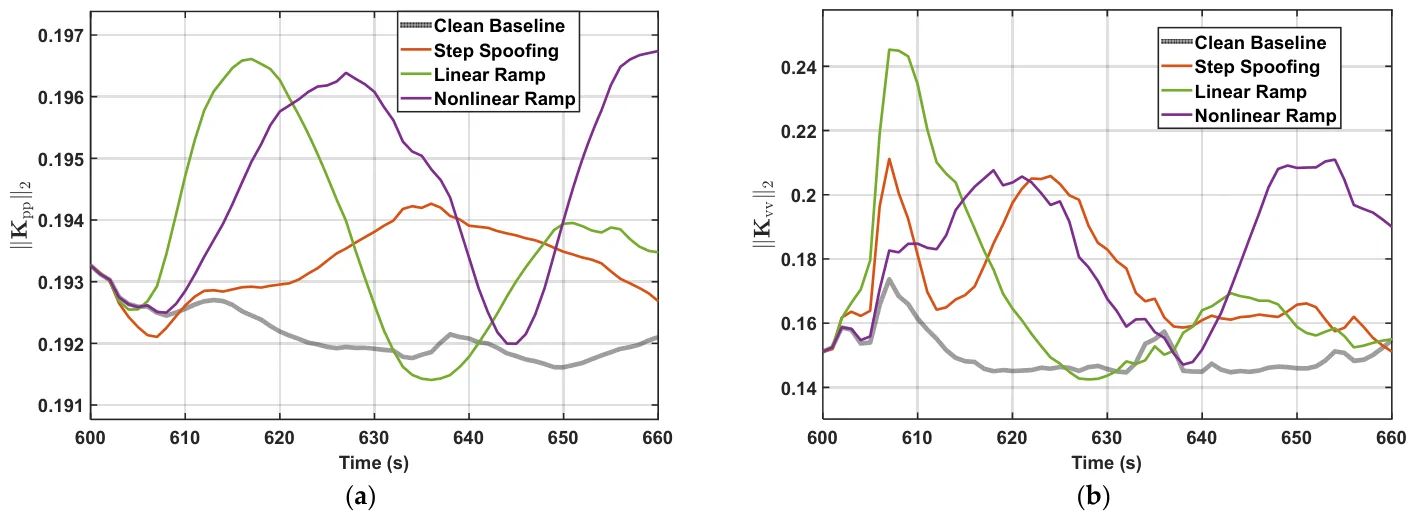
Dynamic evolution comparison of primary gain magnitudes (L2-norm) under different spoofing scenarios: (**a**) Position primary gain $K_{pp}$ magnitude response; (**b**) Velocity primary gain $K_{vv}$ magnitude response.

The primary gains primarily characterize the direct update weighting of measurements on their corresponding identical state dimensions, including the gain $K_{pp}$ from position measurements to position states, as well as the gain $K_{vv}$ from velocity measurements to velocity states. To comprehensively characterize the overall spatial perturbation magnitude of the gains, the Euclidean norm (L2-norm) is introduced to project the gain vector into its magnitude, defined as follows:

(23) $$\|K{\|}_{2}=\sqrt{K_{x}^{2}+K_{y}^{2}+K_{z}^{2}}$$

where $K_{x}$, $K_{y}$, and $K_{z}$ denote the three orthogonal components of the corresponding gain matrix block. The dynamic evolution of the primary gain magnitudes under different spoofing scenarios is illustrated in Figure 8.

As can be seen from Figure 8, the magnitudes of both the position primary gain $K_{pp}$ and the velocity primary gain $K_{vv}$ exhibit prominent dynamic fluctuation responses across various spoofing scenarios. To further quantitatively evaluate the perturbation degree of the primary gains, Table 4 summarizes the ASNR metrics for individual components of the primary gains under different spoofing scenarios.

2. Position Coupling Gains

**Figure 9:**
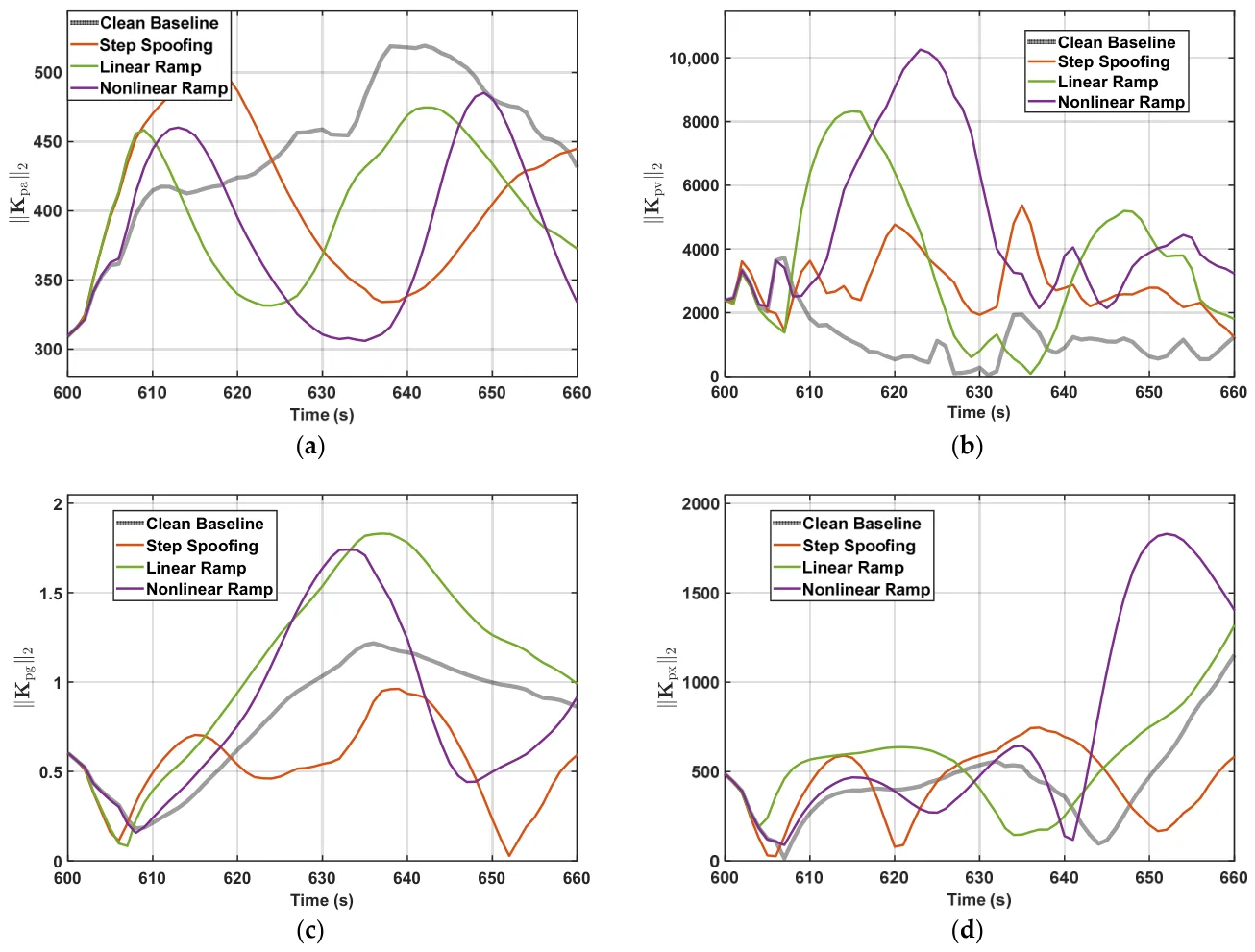
Dynamic evolution comparison of position coupling gain magnitudes ($L_{2}$-norm) under different spoofing scenarios: (**a**) Position-attitude coupling gain $K_{pa}$ magnitude response; (**b**) Position-velocity coupling gain $K_{pv}$ magnitude response; (**c**) Position-gyroscope bias coupling gain $K_{pg}$ magnitude response; (**d**) Position-accelerometer bias coupling gain $K_{px}$ magnitude response.

The position coupling gains reflect the cross-update weighting of position measurement information on other non-position state dimensions, including attitude, velocity, and sensor biases. Specifically, these comprise the gain mapped to attitude $K_{pa}$, the gain mapped to velocity $K_{pv}$, the gain mapped to gyroscope bias $K_{pg}$, and the gain mapped to accelerometer bias $K_{px}$. Given that position measurements typically serve as the most direct target of GNSS spoofing interference, the sensitivity of such coupling gains to spoofing attacks is of paramount significance. The dynamic evolution of the magnitudes of individual position coupling gains under different spoofing scenarios is illustrated in Figure 9.

As demonstrated in Figure 9, the injection of spoofing signals disrupts the steady-state equilibrium of the Kalman filter, causing all four categories of position coupling gains to deviate from the clean baseline and exhibit severe dynamic oscillations. Table 5 presents the quantitative ASNR statistical results of the position coupling gains across various spoofing scenarios.

3. Velocity Coupling Gains

The velocity coupling gains reflect the cross-update weighting of velocity measurement information on other non-velocity state dimensions, such as attitude, position, and inertial sensor biases. Specifically, these comprise the gain mapped to attitude $K_{va}$, the gain mapped to position $K_{vp}$, the gain mapped to gyroscope bias $K_{vg}$, and the gain mapped to accelerometer bias $K_{vx}$.

**Figure 10:**
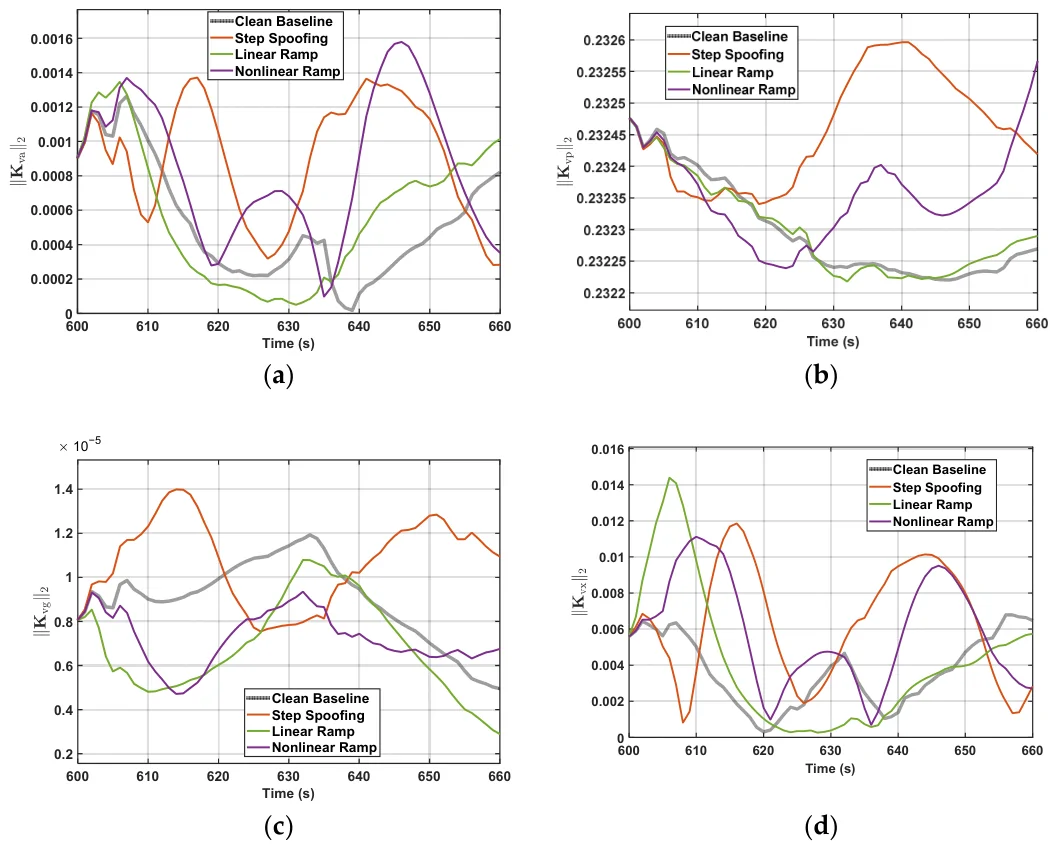
Dynamic evolution comparison of velocity coupling gain magnitudes ($L_{2}$-norm) under different spoofing scenarios: (**a**) Velocity-attitude coupling gain $K_{va}$ magnitude response; (**b**) Velocity-position coupling gain $K_{vp}$ magnitude response; (**c**) Velocity-gyroscope bias coupling gain $K_{vg}$ magnitude response; (**d**) Velocity-accelerometer bias coupling gain $K_{vx}$ magnitude response.

As can be seen from Figure 10, the state divergence induced by GNSS spoofing interference disrupts the steady-state gain structure of the filter, causing various velocity coupling gains to exhibit varying degrees of anomalous responses. Table 6 details the ASNR metrics of the velocity coupling gains across different scenarios, which are utilized to evaluate the error propagation effect triggered by the interference within the velocity measurement channels.

Synthesizing the evolution processes and statistical data of the primary and coupling gain components discussed above, it is evident that the components of the Kalman gain matrix experience varying degrees of perturbation across different GNSS spoofing scenarios compared to the spoofing-free baseline. A deeper comparative analysis of the dynamic response processes and the ASNR statistical metrics leads to two key conclusions:

- Dynamic Phase Lag in Response Timeliness: The perturbation of the gain matrix relies on the progressive accumulation and propagation of state errors, resulting in a pronounced phase lag relative to the onset of spoofing injection. Consequently, it fails to achieve rapid and instantaneous alarms.
- Weak Sensitivity to Magnitude Perturbations: As indicated by the statistical data, the gain in ASNR improvements across all scenarios remains relatively limited, showing no significant order-of-magnitude surge.

Therefore, although spoofing interference can induce alterations in the filtering gain, it remains impractical in actual engineering applications to rely solely on the magnitude fluctuations of the Kalman gain as an independent and effective metric for detecting or identifying GNSS spoofing attacks.

#### 4.2.3. Analysis of the Impact of GNSS Spoofing Interference on Error State Quantities

Based on the preceding analysis of Kalman gain characteristics, GNSS spoofing interference exerts minimal impact on the steady-state numerical values of the gain matrix $K_{k}$, keeping $K_{k}$ within a narrow fluctuation range. In Kalman filtering theory, the standard discrete state-estimation measurement update equation, derived from Equation (14), is given by:

(24) $${\hat{X}}_{k}={\hat{X}}_{k|k-1}+K_{k}(Z_{k}-H_{k}{\hat{X}}_{k|k-1})$$

Under the closed-loop feedback correction framework, the error state estimate from the previous epoch ${\hat{X}}_{k-1}$ is typically reset to zero. Consequently, prior to the arrival of the measurement at the next epoch, the one-step a priori prediction of the error state identically satisfies:

(25) $${\hat{X}}_{k|k-1}=\Phi_{k|k-1}{\hat{X}}_{k-1}=0$$

Substituting ${\hat{X}}_{k|k-1}=0$ into the measurement update equation directly simplifies it to:

(26) $${\hat{X}}_{k}=K_{k}\delta Z_{k}$$

As can be seen, under the mapping of the quasi-constant gain matrix $K_{k}$, the error state estimate ${\hat{X}}_{k}$ across all dimensions is essentially a linear projection of the measurement innovation $\delta Z_{k}$ allocated by the gain matrix. Therefore, its dynamic evolution remains highly synchronized with that of the innovation sequence.

**Figure 11:**
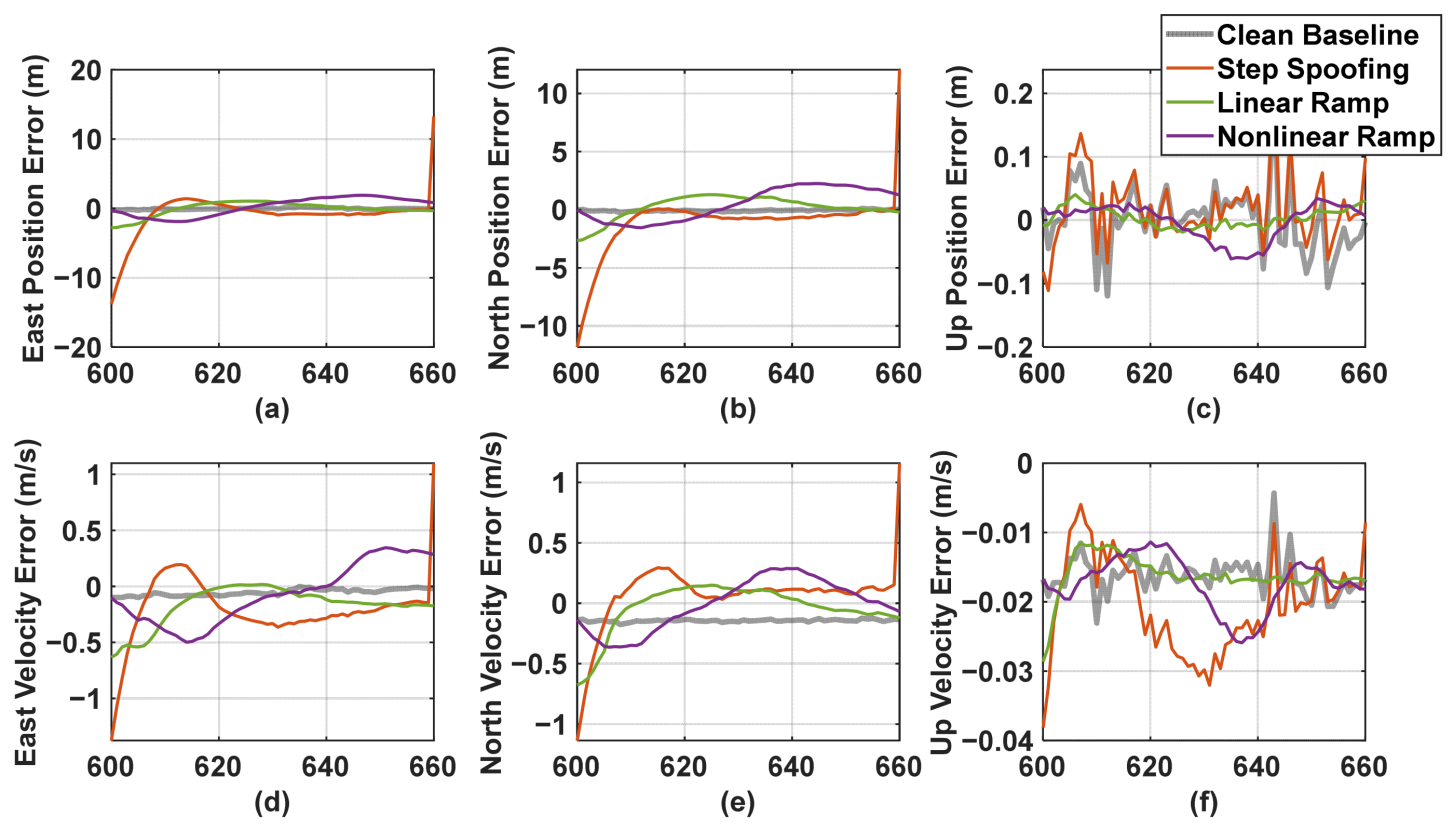
Dynamic comparison of position and velocity error states under different spoofing scenarios: (**a**) East position error; (**b**) North position error; (**c**) Up position error; (**d**) East velocity error; (**e**) North velocity error; (**f**) Up velocity error.

First, regarding the position and velocity error states that characterize spatial kinematics, their dynamic responses are illustrated in Figure 11.

**Figure 12:**
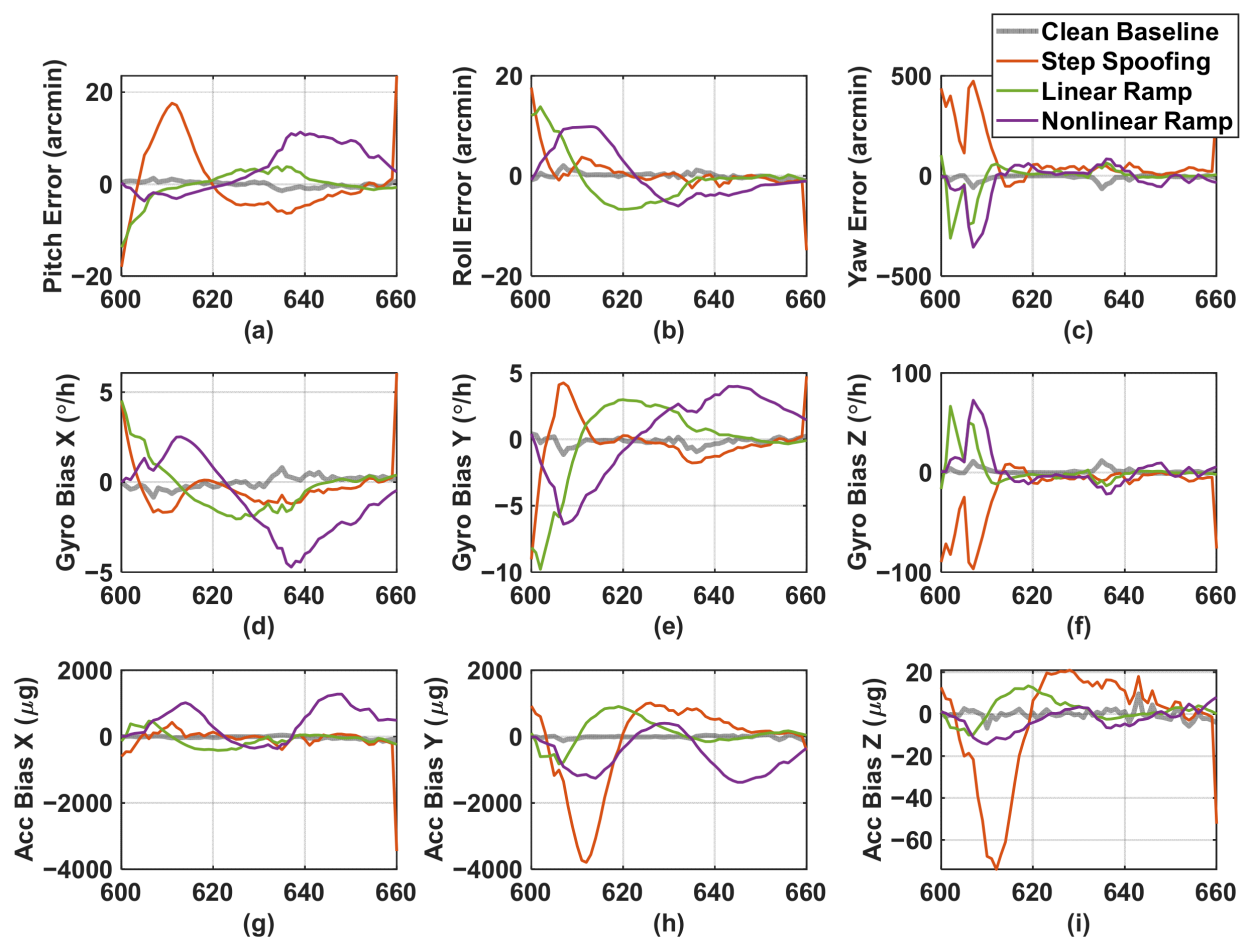
Dynamic comparison of attitude and sensor bias error states under different spoofing scenarios: (**a**) Pitch error; (**b**) Roll error; (**c**) Yaw error; (**d**) Gyroscope bias X-axis; (**e**) Gyroscope bias Y-axis; (**f**) Gyroscope bias Z-axis; (**g**) Accelerometer bias X-axis; (**h**) Accelerometer bias Y-axis; (**i**) Accelerometer bias Z-axis.

Second, regarding the inertial sensor error states that characterize the internal inertial reference, their dynamic responses are illustrated in Figure 12.

Table 7 provides a detailed summary of the ASNR metrics for the error states across the clean baseline and the three spoofing scenarios within the statistical interval of 600 s to 660 s. Through this cross-sectional comparison, the specific disruption level of spoofing energy across different state dimensions during the attack duration is distinctly demonstrated.

Combining the statistical results from Figure 11 and Figure 12 with Table 7, the following two key conclusions can be drawn:

- Amplitude Attenuation in Kinematic Error State Responses: GNSS spoofing has a severe impact on the directly measured position and velocity error states. However, compared with the measurement innovations, the anomalous response amplitudes of the position and velocity error states exhibit pronounced attenuation characteristics upon being mapped through the Kalman gain matrix. The maximum ASNR reaches only 116.3291, which is substantially lower than the corresponding innovation peak of 341.4181.
- Moderate Impact on Inertial Sensor Error States: GNSS spoofing not only exerts a noticeable influence on the directly measured position and velocity error states, but also simultaneously perturbs the unmeasured attitude errors, gyroscope bias corrections, and accelerometer bias corrections. This indicates that external false measurements rapidly diffuse toward the inertial reference via the cross-coupling of the filtering gains; nevertheless, the overall extent of this impact remains moderate, with a maximum ASNR of only 38.7745.

#### 4.2.4. Analysis of the Impact of GNSS Spoofing Interference on Covariance

Within the Kalman filtering framework, the a posteriori error covariance matrix $P_{k}$ serves as the core metric for quantifying system state estimation uncertainty. Its magnitude directly reflects the filter’s confidence level in its own estimation results and constitutes a vital parameter for evaluating system robustness and anti-interference capability. The standard equations for covariance one-step prediction and measurement update are given as follows:

(27) $$\left\{\begin{array}{l} P_{k|k-1}=\Phi_{k,k-1}P_{k-1}\Phi_{k,k-1}^{T}+Q_{k-1}\\ P_{k}=(I-K_{k}H_{k})P_{k|k-1} \end{array}\right.$$

As can be seen from the above formulation, the recursive iteration of the covariance matrix does not directly incorporate the measurement innovations; rather, it is primarily governed by the process noise covariance matrix Q, the measurement noise covariance matrix R (implicitly embedded within the gain $K_{k}$), and the state transition matrix $\Phi_{k,k-1}$ determined by the current operating states. To quantify the ultimate influence on filtering confidence caused by minor perturbations in the Φ matrix—which are induced by spoofing-induced state deviations—Table 8 details the ASNR metrics of the standard deviations corresponding to the main diagonal of individual state components under the interference-free baseline and three typical spoofing scenarios.

According to the synthesized ASNR statistical data, across different GNSS spoofing scenarios, the standard deviations of the Kalman filter a posteriori covariance matrix $P_{k}$ exhibit negligible variations compared with the interference-free baseline. The ASNR magnitudes remain highly consistent between directly observed states (such as position and velocity) and weakly observable states (such as attitude and inertial sensor biases). This indicates that GNSS spoofing interference fails to induce an adaptive expansion of the filter’s internal covariance, causing the system to maintain its nominal estimation confidence despite a sharp surge in actual errors. Consequently, it can be concluded that the magnitude variation characteristics of the covariance matrix alone cannot effectively detect such spoofing attacks.

Synthesizing the in-depth analyses across the four core dimensions—measurement innovation, filtering gain, error states, and state covariance matrix—it is evident that within GNSS/INS integrated navigation systems, the filtering innovation sequence serves as the most sensitive and effective statistical metric for characterizing and detecting spoofing interference. This is primarily attributed to the following three reasons:

- Rapid Response Timeliness: GNSS spoofing attacks essentially involve the direct tampering of the system measurement domain. As the discrepancy between actual measurements and one-step state predictions, the filtering innovation resides at the forefront of information fusion, enabling it to capture externally injected anomalous biases in the most direct and sensitive manner.
- Severe Perturbation Intensity: A comparative evaluation of the ASNR statistical metrics reveals that during spoofing events, the magnitude signal-to-noise ratio of the filtering innovation exhibits an order-of-magnitude leap, peaking at 341.4181. This value far exceeds the faint responses observed in the filtering gains (maximum: 22.6023), error states (maximum: 116.3291), and covariance matrix (maximum: 19.3014), thereby demonstrating exceptional sensitivity to anomalies.
- Well-Behaved Statistical Properties: Under nominal, interference-free operating conditions, the filtering innovation sequence strictly follows a zero-mean Gaussian white noise distribution. This well-behaved statistical characteristic provides an ideal reference baseline for establishing rigorous theoretical detection thresholds.

Therefore, developing GNSS spoofing detection mechanisms based on the filtering innovation sequence is generally recognized as a highly reliable and efficient technical approach in engineering practice.

### 4.3. Impact and Constraints of IMU Accuracy on Innovation Response

The preceding analysis demonstrates that the filtering innovation sequence serves as an effective feature metric for GNSS spoofing detection. In the measurement update phase of the integrated INS/GNSS navigation system, the innovation essentially characterizes the discrepancy between external GNSS observations and internal, standalone INS dead-reckoning states. The confidence of the one-step INS state predictions—along with its resistance to being pulled by external anomalous measurements—is strictly constrained by the hardware grade of the IMU. A high-grade IMU features extremely low intrinsic drift, enabling it to maintain high-confidence inertial dead reckoning during anomalous measurement injections and thereby eliciting pronounced innovation spikes. Conversely, low-to-medium-cost MEMS IMUs exhibit rapid error divergence, making the integrated system more susceptible to smooth pulling by false measurements during the filtering fusion phase, which consequently dampens the anomalous response magnitude of the innovation. Therefore, to comprehensively evaluate the generalizability of innovation-based spoofing detection mechanisms across practical engineering scenarios, it is imperative to investigate the specific constraint mechanisms imposed by different IMU accuracy grades on the innovation response characteristics.

To decouple the independent mechanisms and combined constraint patterns of Angle Random Walk (ARW) and Velocity Random Walk (VRW) on innovation response characteristics, three controlled experimental groups are designed: Group A (A1–A3) varies solely the gyroscope ARW while holding the accelerometer VRW at a high-grade baseline; Group B (B1–B3) varies solely the accelerometer VRW while keeping the gyroscope ARW at a high-grade baseline; and Group C (C1–C3) concurrently degrades both ARW and VRW from high to low grades. The detailed parameter configurations of random walk noises for the IMUs across different grades are summarized in Table 9.

According to Equation (22), when the evaluated parameter is the innovation $\delta Z_{k}$, its ASNR calculation formula simplifies to:

(28) $$\mathrm{ASNR}=\frac{\max|\delta Z_{k}|}{\sigma_{clean}},\;\;\;\;k\in [k_{s},k_{e}]$$

where $k_{s}$ and $k_{e}$ denote the discrete sampling epochs corresponding to the onset and termination of spoofing interference, respectively, and $\sigma_{clean}$ represents the steady-state standard deviation of the innovation component under interference-free conditions.

From Equation (21), the innovation covariance matrix satisfies $S_{k}=H_{k}P_{k|k-1}H_{k}^{T}+R_{k}$. As the integrated navigation system reaches steady state, the a priori error covariance matrix $P_{k|k-1}$ tends toward a constant convergence matrix $P_{\infty }$, and the innovation covariance matrix $S_{k}$ likewise converges to a time-invariant, steady-state constant matrix $S_{\infty }$. Extracting the i diagonal element and taking the square root yields the standard deviation for the i-th channel:

(29) $$\sigma_{clean,i}=\sqrt{{\left[S_{\infty }\right]}_{ii}}=\sqrt{{\left[HP_{\infty }H^{T}+R\right]}_{ii}}$$

where H and R denote the steady-state observation matrix and measurement noise covariance matrix of the system, respectively, and ${\left[\;\cdot \;\right]}_{ii}$ denotes the extraction of the i-th diagonal element of a matrix. From the discrete state covariance propagation equation $P_{k+1}=\Phi P_{k}\Phi^{T}+Q$, it is known that under steady-state filtering, $P_{\infty }$ is directly constrained by the process noise covariance Q. In practical engineering and simulation evaluations, the three-axis hardware noise of a given IMU model is typically isotropic. Letting the three-axis nominal ARW parameter be uniformly denoted by $\sigma_{arw}$ and the nominal VRW parameter by $\sigma_{vrw}$, the baseline steady-state variance of the i-th channel innovation can be approximated as a linear superposition of the GNSS measurement noise floor variance and the IMU random walk variances:

(30) $$\sigma_{clean,i}^{2}\approx \sigma_{0,i}^{2}+c_{1,i}\cdot \sigma_{arw}^{2}+c_{2,i}\cdot \sigma_{vrw}^{2}$$

where $\sigma_{0,i}^{2}={\left[R\right]}_{ii}$ is the system asymptotic noise floor variance determined solely by GNSS measurement noise, and $c_{1,i}$ and $c_{2,i}$ are transfer scale coefficients that account for inertial error integration propagation and dimensional unification. Substituting Equation (30) into Equation (28) establishes the theoretical analytical fitting model for the i-th dimensional innovation ASNR with respect to the IMU random walk parameters:

(31) $$\mathrm{ASNR}(\mathrm{ARW},\mathrm{VRW})=\frac{\max|\delta Z_{k,i}|}{\sqrt{\sigma_{0}^{2}+c_{1}\cdot \sigma_{arw}^{2}+c_{2}\cdot \sigma_{vrw}^{2}}},\;\;\;\;k\in [k_{s},k_{e}]$$

where $\delta Z_{k,i}$ denotes the i-th channel innovation at the k-th epoch. As indicated by Equation (31), the ASNR exhibits a negative correlation with the intrinsic noise level of the IMU. With the progressive increase in sensor ARW and VRW, the baseline noise floor of the innovation is passively amplified, which leads to a nonlinear degradation in the system’s response sensitivity to spoofing perturbations.

To validate the efficacy of the aforementioned theoretical fitting model in realistic dynamic scenarios, evaluations are first conducted under the step spoofing scenario. Across the nine IMU accuracy configurations defined in Table 9, the ASNR statistical results for the 6-dimensional Kalman filtering innovation components are summarized in Table 10.

From the statistical data in Table 10, it can be observed that under step spoofing interference, the innovation response exhibits the following prominent characteristics:

- Exceptionally Strong Position Innovation Response: The East and North position innovations instantaneously excite massive immediate residuals, with their ASNR values generally reaching an extremely high level of 260 to 510.
- Degradation of IMU Accuracy Induces Significant Deterioration in Velocity Innovation Response: Comparing the evolution trends among Groups A, B, and C reveals that as the IMU hardware accuracy progressively deteriorates from high (A1/B1/C1) to low (A3/B3/C3), the horizontal velocity innovation ASNR exhibits a monotonic attenuation trend (decreasing from 47.64 to 23.99 in the East direction and from 46.48 to 21.56 in the North direction, with a drop of approximately 50%). The underlying mechanism is that the increased random walk noise in low-grade IMUs elevates the steady-state baseline noise variance $\sigma_{\mathrm{clean}}$ during nominal filter operation, thereby suppressing the relative signal-to-noise ratio gain induced by step perturbations.
- Position Innovation Exhibits Strong Robustness to Hardware Noise: Compared with velocity innovations, the horizontal position innovation ASNR remains at a high level of several hundred across all accuracy grades without exhibiting noticeable step-like degradation. This indicates that the magnitude of the position step far exceeds the sensor’s own noise diffusion level, enabling position innovations to stably maintain high-sensitivity capture capability against step spoofing across different hardware configurations.

To further evaluate the more covert linear and nonlinear ramp spoofing scenarios, the ASNR statistical results of the 6-dimensional Kalman filtering innovation components across the nine IMU accuracy configurations are summarized in Table 11 and Table 12, respectively.

Comparing the statistical data in Table 11 and Table 12 reveals the following core evolution patterns and hardware constraint mechanisms:

- Linear Ramp Scenario Dominated by Horizontal Velocity Innovation Responses, with Position Innovations Severely Constrained by IMU Accuracy: Under linear ramp spoofing, the continuous injection of a constant velocity bias induces significant anomalous responses in horizontal velocity innovations, with the East and North velocity ASNRs maintaining relatively high levels across all groups (41.16–70.46). Conversely, the ASNR of the horizontal position innovation decays markedly as the IMU hardware accuracy degrades.
- Faint Responses of Both Innovation Types Under Nonlinear Ramp Scenarios, Constrained Deeply by IMU Accuracy: Due to the continuous acceleration and smooth variation inherent in the smooth velocity addition profile, the ASNRs of both position and velocity innovations are severely compressed. Taking the East innovation as an example, under the high-grade baseline (C1), the East position and velocity innovation ASNRs reach only 22.0427 and 33.4195, respectively. When the IMU accuracy further degrades to the low-grade configuration (C3), the East position and velocity innovation ASNRs decline to 7.6495 and 8.6549, respectively.

Synthesizing the evaluation results across the three spoofing scenarios, the degradation of IMU hardware accuracy primarily acts by elevating the baseline variance of the system’s steady-state innovations, thereby compressing the response level across all dimensions from the bottom up. Under abrupt step spoofing, the position innovation magnitude is massive and exhibits strong robustness against hardware noise, whereas the velocity innovation detection sensitivity displays a monotonic attenuation as IMU accuracy decreases. Under linear ramp spoofing with a constant velocity bias, velocity innovations dominate the response profile; however, increases in ARW and VRW severely blunt the sensitivity of position innovations to gradually accumulating biases. Under highly covert nonlinear ramp scenarios, the smooth profile transition inherently compresses full-dimensional innovation responses; the noise masking effect of low-grade IMUs further depresses the signal-to-noise ratio to near-marginal levels, exposing low-cost MEMS platforms to extremely high risks of missed detection.

In summary, although inertial sensor accuracy does not alter the underlying dynamic response morphology of the system following spoofing injection, it fundamentally dictates the sensitivity lower bound and reliability boundary of conventional innovation detection mechanisms across various attack scenarios. To overcome the inherent vulnerability of low-cost MEMS integrated navigation systems to noise masking under ramp spoofing, it is imperative to introduce moving-window cumulative residual testing and online IMU error adaptive compensation mechanisms, thereby breaking through the physical constraints imposed by low-accuracy hardware on spoofing detection performance.

## 5. Conclusions

By combining theoretical derivations with simulation verifications, this article systematically investigates the operational characteristics and influence mechanisms of typical GNSS spoofing interference on the INS/GNSS integrated navigation Kalman filtering system. Specifically, three spoofing pull-off models (step-type, linear ramp-type, and nonlinear ramp-type) are established, and the error propagation dynamics across four core filtering dimensions are quantitatively dissected using the ASNR metric. Furthermore, the physical constraint laws of IMU accuracy grades on spoofing detection boundaries are revealed. The primary conclusions are drawn as follows:

- Among the four core parameters—measurement innovation, filter gain, error states, and state covariance—the filtering innovation responds most directly and sensitively to spoofing interference. The peak ASNR of the filtering innovation exhibits an order-of-magnitude leap, reaching 341.4181, vastly outperforming the faint responses observed in error states (maximum: 116.3291), filter gains (maximum: 22.6023), and state covariance (maximum: 19.3014). Meanwhile, the filtering innovation possesses the well-behaved statistical property of strictly following a zero-mean Gaussian distribution under nominal conditions, rendering it the most suitable and effective feature metric for spoofing interference detection.
- The hardware accuracy grade of the IMU fundamentally dictates the sensitivity lower bound and reliability boundary of the innovation-based detection mechanism. Under step spoofing, position innovations exhibit strong robustness across all hardware grades (ASNR: 260–510), whereas velocity innovation ASNR drops monotonically by approximately 50% (East: from 47.64 to 23.99; North: from 46.48 to 21.56) due to the elevated baseline noise floor in low-grade IMUs. Under linear ramp spoofing, the constant velocity step stimulates significant velocity innovation responses across all IMU configurations (ASNR: 41.16–70.46); however, with the degradation of IMU accuracy, the position innovation ASNR decays substantially (the minimum horizontal position ASNR drops to only 5.3941). Under nonlinear ramp spoofing, high-grade IMUs can still stimulate detectable innovation conflicts (maximum position and velocity ASNRs of 25.8409 and 33.4195, respectively), whereas the maximum horizontal position and velocity ASNRs of low-cost IMUs fall below 10. This noise masking effect severely compresses the response magnitude, exposing low-cost platforms to an extremely high risk of missed detection.

Future research could further encompass diverse vehicle motion states (such as acceleration, deceleration, and slope variation) and multi-dimensional spoofing directions (such as orthogonal and opposite directions), thereby providing a more systematic and comprehensive theoretical foundation for constructing GNSS anti-spoofing defense schemes.

## Figures and Tables

**Table 1:** Parameter configuration of typical spoofing interference scenarios.

Spoofing Type	GNSS Spoofed Position			GNSS Velocity Setting			Remarks
	East	North	Up	East	North	Up	
Step	$P_{E}(600)+100$	$P_{N}(600)+100$	$P_{D}(600)$	$V_{E}(t)$	$V_{N}(t)$	$V_{U}(t)$	/
Linear Ramp	/	/	/	$V_{E}(t_{s})+5$	$V_{N}(t_{s})+5$	0	Constant velocity
Nonlinear Ramp	/	/	/	$V_{E}(t_{s})+7.85$	$V_{N}(t_{s})+7.85$	0	Terminal velocity

**Table 2:** Sensor parameters.

Sensor	Noise Metric	Axis	Value
IMU	Angle Random Walk (ARW)/(${}^{\circ}/\sqrt{h}$)	X	0.1056
		Y	0.1093
		Z	0.0617
	Velocity Random Walk (VRW)/($\mu g/\sqrt{\mathrm{Hz}}$)	X	135.5
		Y	133.4
		Z	125.3
GNSS	Position Measurement Standard Deviation/m	East	0.60
		North	0.60
		Up	1.20
	Velocity Measurement Standard Deviation/(m/s)	East	0.08
		North	0.08
		Up	0.12

**Table 3:** ASNR of innovations under various spoofing scenarios.

State Category	State Component	Clean	Step	Linear Ramp	Nonlinear Ramp
Position Innovation	East	2.6055	295.1477	14.9887	18.2422
	North	2.6266	341.4181	15.1505	25.8409
	Up	3.5593	3.0165	0.9381	1.3286
Velocity Innovation	East	2.8914	47.6367	54.3531	22.0427
	North	2.4740	46.4819	59.0785	33.4195
	Up	4.7212	5.2196	0.6962	0.9976

**Table 4:** ASNR of primary gains under different interference scenarios.

State Category	State Component	Clean	Step	Linear Ramp	Nonlinear Ramp
$K_{pp}$	East	2.2626	4.2167	13.2583	10.3088
	North	1.9849	7.3629	2.8824	2.2439
	Up	1.9043	1.9043	1.9043	1.9043
$K_{vv}$	East	4.1185	4.4980	22.6023	13.1153
	North	3.8272	8.6975	5.1401	3.7990
	Up	2.4992	5.6890	2.4992	2.4992

**Table 5:** ASNR of position coupling gains under different interference scenarios.

State Category	State Component	Clean	Step	Linear Ramp	Nonlinear Ramp
$K_{pa}$	East/Pitch	4.2054	7.9000	4.2054	4.2054
	North/Roll	1.8156	1.7836	3.1955	1.8156
	Up/Yaw	1.5615	2.5725	2.4103	1.5423
$K_{pv}$	East	2.8932	8.6474	7.8762	10.7362
	North	3.0520	4.2026	8.0231	9.5964
	Up	2.2389	3.7562	2.2389	2.2389
$K_{pg}$	X-axis	2.4471	4.0667	2.7341	4.4534
	Y-axis	2.4583	2.4583	2.4583	2.4583
	Z-axis	2.3360	3.7523	5.5991	2.3360
$K_{px}$	X-axis	2.2457	1.8791	1.8960	1.4520
	Y-axis	2.7015	1.5581	4.0185	1.3530
	Z-axis	2.1763	2.1763	2.1763	2.1763

**Table 6:** ASNR of velocity coupling gains under different interference scenarios.

State Category	State Component	Clean	Step	Linear Ramp	Nonlinear Ramp
$K_{va}$	East/Pitch	1.7705	4.0965	2.1193	1.4373
	North/Roll	2.2632	3.7153	2.8449	2.2501
	Up/Yaw	1.9633	2.0341	1.7511	1.9622
$K_{vp}$	East	3.0496	4.1993	8.0168	9.5889
	North	2.8900	8.6378	7.8674	10.7243
	Up	2.2390	3.7562	2.2390	2.2390
$K_{vg}$	X-axis	1.8435	2.6203	4.0161	1.1070
	Y-axis	2.4806	3.4822	3.4411	1.2568
	Z-axis	1.9190	2.9348	2.7428	1.9164
$K_{vx}$	X-axis	2.1877	4.0950	4.1753	1.4363
	Y-axis	1.7875	3.9335	2.7213	1.6548
	Z-axis	2.4422	2.4422	2.4422	2.4422

**Table 7:** ASNR of Kalman filter error states under various spoofing scenarios.

State Category	State Component	Clean	Step	Linear Ramp	Nonlinear Ramp
Position	East	2.8000	116.3291	17.9845	15.9578
	North	2.3626	79.2990	17.7909	12.7183
	Up	3.9338	4.7063	1.0187	3.2263
Velocity	East	3.2841	69.6018	21.6646	15.5736
	North	2.7612	42.5011	20.7923	12.4999
	Up	4.6250	5.6939	2.6741	5.6216
Attitude	Pitch	2.2806	37.3298	21.7491	17.9195
	Roll	3.3065	29.4629	23.0100	16.4511
	Yaw	3.9453	34.0928	21.4650	24.6429
Gyroscope Zero Bias	X-axis	2.6019	18.6757	13.9656	14.5638
	Y-axis	3.2762	29.7202	32.2523	20.8693
	Z-axis	3.8466	35.9281	23.8659	26.0528
Accelerometer Zero Bias	X-axis	2.8899	35.1769	12.3113	32.5309
	Y-axis	3.6093	38.7745	21.5432	32.0210
	Z-axis	4.0355	30.3157	5.6082	5.8050

**Table 8:** ASNR of standard deviations of a posteriori state covariance under different interference scenarios.

State Category	State Component	Clean	Step	Linear Ramp	Nonlinear Ramp
Position	East	2.2175	4.1262	12.8874	10.0419
	North	1.9497	7.1950	2.8285	2.2034
	Up	1.9043	1.9043	1.9043	1.9043
Velocity	East	4.0489	4.4058	19.3014	11.9504
	North	3.7376	7.9210	4.9175	3.7118
	Up	2.4988	5.6840	2.4988	2.4988
Attitude	Pitch	1.6542	1.7977	1.9846	3.4553
	Roll	1.8801	3.3132	4.4525	1.8577
	Yaw	2.1176	8.3323	8.8438	7.0516
Gyroscope Zero Bias	X-axis	2.5879	3.0578	5.8397	2.9733
	Y-axis	2.9804	4.6865	8.6153	6.7030
	Z-axis	2.0608	6.1957	5.6901	5.0054
Accelerometer Zero Bias	X-axis	1.5425	1.5425	2.3318	1.5451
	Y-axis	1.6495	1.3761	1.8380	1.6985
	Z-axis	1.5811	1.5006	1.7625	1.6313

**Table 9:** Parameter configuration of random walks for IMUs with different accuracy grades.

Group	ARW/(Deg/Sqrt(h))			VRW/(Ug/Sqrt(Hz))		
	X	Y	Z	X	Y	Z
A1	0.1056	0.1093	0.0617	135.5	133.4	125.3
A2	0.5569	0.5413	0.5309			
A3	2.0000	2.0000	2.0000			
B1	0.1056	0.1093	0.0617	135.5	133.4	125.3
B2				546.1	540.9	549.9
B3				2000.0	2000.0	2000.0
C1	0.1056	0.1093	0.0617	135.5	133.4	125.3
C2	0.5569	0.5413	0.5309	546.1	540.9	549.9
C3	2.0000	2.0000	2.0000	2000.0	2000.0	2000.0

**Table 10:** Statistical comparison of innovation ASNR under different IMU accuracy configurations in the step spoofing scenario.

Group	Position Innovation ASNR			Velocity Innovation ASNR		
	East	North	Up	East	North	Up
A1	295.1477	341.4181	3.0165	47.6367	46.4819	5.2196
A2	273.6977	453.2400	12.7447	33.9037	25.6898	10.0105
A3	311.8926	503.3740	3.8815	28.4203	23.1604	4.9542
B1	295.1477	341.4181	3.0165	47.6367	46.4819	5.2196
B2	265.1410	393.3976	3.4398	31.7893	30.5634	4.9965
B3	308.5641	477.5494	3.3791	34.0560	24.5627	4.5978
C1	295.1477	341.4181	3.0165	47.6367	46.4819	5.2196
C2	327.5399	507.2693	3.4367	37.8965	36.4745	4.9687
C3	306.2116	500.2943	3.3859	23.9903	21.5614	4.5804

**Table 11:** Statistical comparison of innovation ASNR under different IMU accuracy configurations in the linear ramp spoofing scenario.

Group	Position Innovation ASNR			Velocity Innovation ASNR		
	East	North	Up	East	North	Up
A1	14.9883	15.1505	0.9381	54.3531	59.0785	0.6962
A2	3.8935	7.6825	1.2634	44.7950	50.7219	1.3053
A3	5.4555	8.5576	0.6338	60.5562	68.1396	2.1581
B1	14.9883	15.1505	0.9381	54.3531	59.0785	0.6962
B2	7.6160	12.3594	0.2280	41.1635	43.3937	0.5094
B3	1.3490	2.5912	0.3626	66.2247	66.6608	0.5637
C1	14.9883	15.1505	0.9381	54.3531	59.0785	0.6962
C2	5.0158	5.1890	0.4211	54.9885	70.4626	0.8384
C3	5.3941	7.9267	0.3525	61.7013	67.4169	0.9857

**Table 12:** Statistical comparison of innovation ASNR under different IMU accuracy configurations in the nonlinear ramp spoofing scenario.

Group	Position Innovation ASNR			Velocity Innovation ASNR		
	East	North	Up	East	North	Up
A1	18.2422	25.8409	1.3286	22.0427	33.4195	0.9976
A2	4.9247	8.7409	0.7650	10.7878	12.6443	0.8006
A3	4.3400	6.9330	1.8281	7.9436	9.7572	1.4751
B1	18.2422	25.8409	1.3286	22.0427	33.4195	0.9976
B2	13.0367	20.9160	0.2009	14.2375	15.2127	0.5316
B3	6.8957	11.3942	0.3626	13.8061	12.7560	0.5585
C1	18.2422	25.8409	1.3286	22.0427	33.4195	0.9976
C2	5.8679	10.2893	0.3376	11.1497	16.4309	0.7327
C3	4.3703	7.1112	0.3525	7.6495	8.6549	0.8572

## Data Availability

Publicly available datasets were analyzed in this study. These data (data_cpt) can be found in the GINav repository at https://github.com/kaichen686/GINav (accessed on 21 August 2026).

## Abbreviations

The following abbreviations are used in this manuscript:

GNSS	Global Navigation Satellite System
INS	Inertial Navigation System
IMU	Kalman Filter
KF	Linear Dichroism
ARW	Angle Random Walk
VRW	Velocity Random Walk
